# The grape berry methylome reveals tissue-specific features associated with metabolism in ripening

**DOI:** 10.1093/hr/uhaf238

**Published:** 2025-09-17

**Authors:** Junhua Kong, Yu Ma, Huan Huang, Virginie Garcia, Qingfeng Niu, Ghislaine Hilbert-Masson, Linda Stammitti, Siqun Wu, Yonglei Yu, Philippe Gallusci, Zhaobo Lang

**Affiliations:** UMR EGFV, Université Bordeaux, Bordeaux Sciences Agro, INRAE, ISVV, Villenave d’Ornon, France; Institute of Advanced Biotechnology, Institute of Homeostatic Medicine, and School of Medicine, Southern University of Science and Technology, Shenzhen 518055, China; CAS Key Laboratory of Tissue Microenvironment and Tumor, Shanghai Institute of Nutrition and Health, Chinese Academy of Sciences, Shanghai 200030, China; UMR EGFV, Université Bordeaux, Bordeaux Sciences Agro, INRAE, ISVV, Villenave d’Ornon, France; National Engineering Laboratory of Crop Stress Resistance Breeding, School of Life Sciences, Anhui Agricultural University, Hefei 230036, China; Advanced Academy, Research Centre for Biological Breeding Technology, Anhui Agricultural University, Hefei, Anhui 230036, China; UMR EGFV, Université Bordeaux, Bordeaux Sciences Agro, INRAE, ISVV, Villenave d’Ornon, France; UMR EGFV, Université Bordeaux, Bordeaux Sciences Agro, INRAE, ISVV, Villenave d’Ornon, France; Institute of Advanced Biotechnology, Institute of Homeostatic Medicine, and School of Medicine, Southern University of Science and Technology, Shenzhen 518055, China; Institute of Advanced Biotechnology, Institute of Homeostatic Medicine, and School of Medicine, Southern University of Science and Technology, Shenzhen 518055, China; UMR EGFV, Université Bordeaux, Bordeaux Sciences Agro, INRAE, ISVV, Villenave d’Ornon, France; Institute of Advanced Biotechnology, Institute of Homeostatic Medicine, and School of Medicine, Southern University of Science and Technology, Shenzhen 518055, China

## Abstract

DNA methylation is a critical epigenetic regulator in plant development, yet its role in grape berry ripening remains poorly understood. Here, we profiled the genome-wide DNA methylation landscapes of two cultivars, ‘Wink’ and ‘Cabernet Sauvignon’, across developmental stages and tissues (skin and pulp tissues), revealing widespread DNA hypermethylation during ripening. We observed a progressive increase in global DNA methylation, particularly in the CHH context, across transposable elements, gene bodies, and adjacent regions during ripening. This hypermethylation was conserved across both varieties and was pronounced in both skin and pulp tissues. Differentially methylated regions (DMRs) revealed tissue-specific methylation patterns, with skin and pulp exhibiting distinct hypermethylation dynamics. Further analysis demonstrated that these tissue-specific hypermethylation dynamics are partially attributable to pre-existing methylation differences between skin and pulp at earlier developmental stages. Functional analysis demonstrated that DNA methylation inhibitors (zebularine and RG108) delayed berry ripening *in vitro*, underscoring the critical role of methylation in this process. Furthermore, RNA-seq analysis identified tissue-specific gene expression changes associated with differential methylation, particularly in metabolic pathways such as anthocyanin biosynthesis, fructose metabolism, and glycolysis. Notably, tissue-specific hypermethylation of genes involved in anthocyanin metabolism correlated with their expression patterns, suggesting a regulatory role for DNA methylation in metabolite accumulation during ripening. Collectively, these findings underscore DNA methylation as a critical regulatory layer that orchestrates tissue-specific gene expression with metabolic shifts during grape maturation, thereby advancing our understanding of epigenetic control in fruit development.

## Introduction

Fleshy fruits have evolved into many different forms to attract foraging animals in exchange for seed dispersal. Based on ripening processes, fruits are generally categorized as either climacteric, such as tomato, banana, and apple, or nonclimacteric fruits, as seen in grape, strawberry, and orange. The defining characteristic of climacteric ripening is a sharp spike in respiration accompanied by a burst of ethylene synthesis at its initiation, a feature absent in nonclimacteric fruits [1]. Most studies of fruit ripening have been carried out with climacteric model tomato, but there is a significant knowledge gap concerning the molecular control of ripening, particularly within nonclimacteric fruits, which have been studied less extensively [2–4].

In both climacteric and nonclimacteric fruits, the ripening program is orchestrated by key factors, including hormones and transcription factors (TFs) [5]. In addition to these canonical regulators, epigenetic modifications, most notably DNA methylation, are also critical for this process [6–8]. DNA methylation functions as a key epigenetic modification implicated in a broad spectrum of biological processes, including genome integrity, gene imprinting, aging, plant development, and stress responses [9, 10]. In plants, cytosine DNA methylation is established through the RNA-directed DNA methylation (RdDM) pathway, whereby 24 nt siRNAs are produced and used to guide DNA methyltransferases to specific targets in the genome. Once established, the inheritance of these methylation marks is ensured by context-dependent maintenance pathways: methylation within symmetrical sequence contexts, specifically CG and CHG (where H denotes A, C, or T), is sustained by the METHYLTRANSFERASE 1 (MET1) and CHROMOMETHYLASE 3 (CMT3) enzymes, respectively; and the maintenance of CHH methylation involves CMT2 and the RdDM pathway [10]. These pathways participate in ripening control in several fruits. In strawberry, the decrease in DNA methylation, which is essential for ripening induction, is due to the reduced activity of the RdDM pathway [11]. Tomato represents another fruit where the ripening process is linked to a reduction in global DNA methylation. However, in this case, a DNA-demethylase gene, *SlDML2*, is upregulated, leading to a widespread reduction in DNA methylation [2–4]. The critical role of *SlDML2* is confirmed by experiments showing that its suppression or knockout results in delayed ripening [2, 3]. In contrast, in sweet orange, DNA methylation increases during ripening, and this is associated with downregulation of *DML* genes. Moreover, treatment with a DNA methylation inhibitor has been shown to disrupt the normal ripening program in oranges [12]. These studies affirm the pivotal role of DNA methylation dynamics in the ripening of fleshy fruits.

Grapevine (*Vitis vinifera* L.) bears a typical nonclimacteric fleshy berry with a vast market demand and economic value for both fresh fruit and processed products such as wine and grapeseed oil, estimated to be worth $US283 billion in 2024 [13]. Studies on ripening of grapes have implications for extending shelf life, optimizing postharvest techniques, reducing loss rates, and breeding high-quality grapevine varieties. Furthermore, the skin and pulp of grape berries have tissue-specific gene expression and metabolic features and undergo different changes during ripening. For instance, accumulation of anthocyanin pigments mainly occurs in the skin, whereas sugar accumulation takes place preferentially in the pulp [14, 15]. Methylomes of grape leaves and berries have been reported [16, 17], but it is very recently that the role of CHH methylation during ripening has been highlighted. An early CHH promoter hypermethylation burst in the ‘Fengzao’ mutant has been reported to silence ~1500 genes—including the ripening repressor JOX1—and accelerate maturation, particularly linking CHH methylation to early ripening phenotypes of grape berries [18]. Abscisic acid (ABA application in ‘Summer Black’ induces DNA methylation change in promoters of genes controlling sugar metabolism, secondary metabolite biosynthesis, ABA homeostasis and stress responses [19]. However, whether and how these methylation dynamics coordinate the broader shifts in gene expression and metabolite profiles during ripening remains to be elucidated. Given the distinct metabolic signatures of grape skin and pulp, characterizing their respective methylomes can deepen our understanding of tissue-specific regulatory programs in grape maturation and the distinct physiological and metabolic changes occurring in these tissues. Moreover, although DNA methylation in fruit tissues has been previously studied, an integrative analysis that connects tissue-specific methylation dynamics with transcriptional and metabolic network reprogramming to systematically uncover regulatory mechanisms during grape ripening is still needed.

Here, we profiled the genome-wide DNA methylation landscapes of ‘Wink’ and ‘Cabernet Sauvignon’ grape berries. Using whole-genome bisulfite sequencing (WGBS), we generated single-base resolution data for three distinct developmental stages: immature, veraison, and ripe. Methylome analysis showed a progressive increase in methylation, particularly in the CHH context, after veraison, with pronounced hypermethylation in promoter regions, transposable elements, and gene bodies. Functional assays using the DNA methyltransferase inhibitors zebularine and RG108 in an *in vitro* ripening system demonstrated concentration-dependent delays in coloration, confirming methylation’s critical role in ripening. RNA-seq and metabolite profiling further linked methylation dynamics to tissue-specific gene expression and metabolic reprogramming. For instance, skin-specific hypermethylation and downregulation of a polyphenol oxidase gene that is involved in anthocyanin degradation coincided with anthocyanin accumulation, while pulp-specific hypermethylation and downregulation of a GL3 TF, a key inducer of the anthocyanin biosynthetic pathway, correlated with the absence of pigmentation. Kyoto Encyclopedia of Genes and Genomes (KEGG) pathway analysis identified methylation-responsive genes enriched in pathways such as anthocyanin biosynthesis, fructose metabolism, and tyrosine degradation, underscoring methylation’s contribution to ripening-associated metabolic changes. Our findings establish that DNA methylation is a conserved, tissue-specific regulator of grape berry ripening, influencing both transcriptional networks and metabolic outcomes. This study provides a more nuanced view of the role that epigenetics plays in the fruit development program.

## Results

### Genome-wide DNA hypermethylation occurs during berry ripening

**Figure 1:**
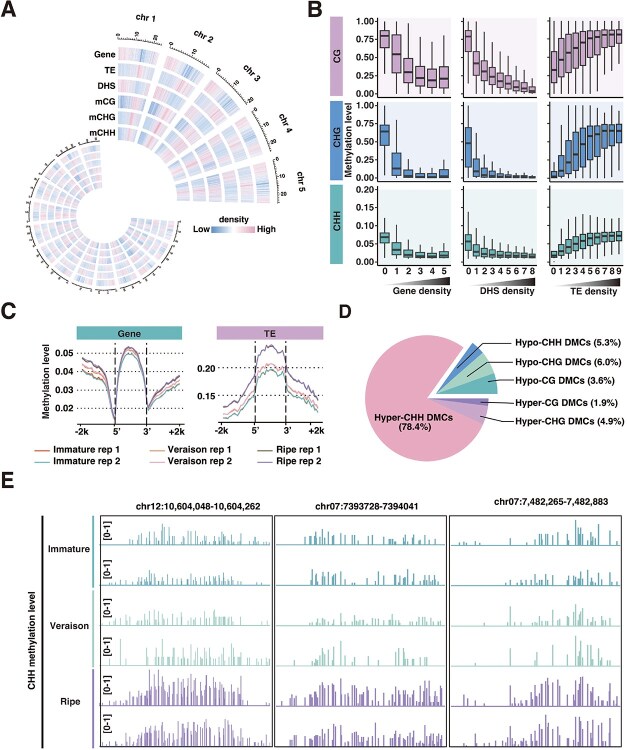
‘Wink’ grape berry undergoes global increase in DNA methylation during ripening. (A) Distribution of genes, transposable elements (TEs), DNase I hypersensitive sites (DHS), and DNA methylation modifications in three contexts. (B) Correlation between DNA methylation level and gene density (left panel), chromatin accessibility (middle panel), and transposable element density (right panel). (C) DNA methylation profiles surrounding genes (left) and transposable elements (right) in samples at immature, veraison, and ripe stages. Two biological replicates are shown. The x-axis indicates the distance relative to the 5′ and 3′ ends of genes or transposable elements, encompassing a 2kb region both upstream and downstream. (D) Percentage of ripening-induced DMCs in ripe compared to immature berries. (E) IGV snapshots showing DNA methylation level profiles in three representative CHH hyper-DMRs. Black boxes highlight the hyper-DMR regions, where increased CHH methylation is observed.

To elucidate the DNA methylation dynamics during grape ripening, we generated high-resolution methylomes for the ‘Wink’ cultivar. Samples representing three distinct developmental stages—immature (IM), veraison (Ver), and ripe (R)—were analyzed in duplicate using WGBS. Sequencing of the WGBS libraries generated a minimum of 97 million 150-bp paired-end reads per sample. The bisulfite conversion rate for all libraries was excellent, exceeding 99.6%. The average sequencing depth was approximately 25×, with valid sites (sequencing depth ≥4) covering 79% of all genomic cytosines (Table S1). High correlation between biological replicates at each stage confirmed data reproducibility (Fig. S1). The average DNA methylation level at immature stage was ~7.2%, roughly one-third that of tomato fruit (22%) [2], with mCG, mCHG, and mCHH levels averaging 37.3%, 16.0%, and 2.7%, respectively. Across chromosomes, methylation, including mCG, mCHG, and mCHH, was enriched in pericentromeric regions, which are typically transposable element-rich and repeat-rich, while methylation tended to be depleted in gene-rich regions (Fig. 1A and B).

Consistent with previous findings in *Arabidopsis* and other plants, cytosine methylation varied across different sequence contexts [20]. In grape berries, CGA context had higher density than CGT, CGC, or CGG contexts, although methylation levels were comparable across CG subcontexts. Among CHG contexts, CAG and CTG contexts present both higher density and higher methylation levels than CCG. For CHH contexts, CCC showed the lowest density, while CAA exhibited the highest, with CAA and CTA showing higher methylation levels than other subcontexts (Fig. S2).

We observed a progressive increase in average DNA methylation level in transposable elements, gene bodies, and adjacent genomic regions (i.e. within 2 kb upstream or downstream) from immature to ripe, with the most pronounced increase occurring after veraison (Fig. 1C). We noticed that promoter regions, transposable element bodies, and flanking regions exhibited pronounced CHH. Further, we identified differentially methylated cytosines (DMCs) in ripe versus immature berries, and found that CHH hyper-DMCs were predominant (78%) among all hyper- and hypo-DMCs (Fig. 1D, Fig. S3A and B) suggesting that ripening-induced hypermethylation is mainly driven by increase in mCHH. Representative hypermethylated regions are illustrated in Fig. 1E. Our discovery of a sustained, genome-wide CHH methylation increase throughout grape berry ripening not only aligns with the early CHH hypermethylation burst in the ‘Fengzao’ mutant [18] but also establishes its conservation in ‘Wink’ and its extension into late ripening stages.

**Figure 2:**
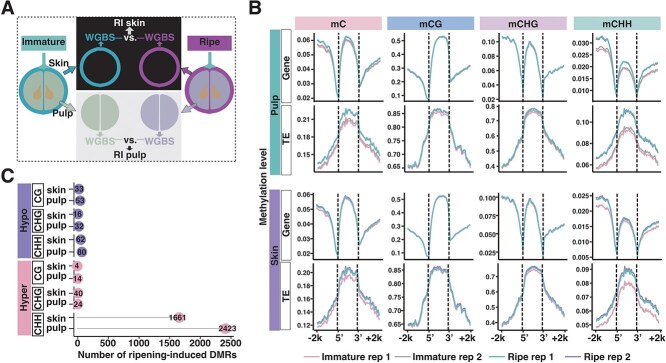
Skin and pulp tissues undergo CHH hypermethylation during ripening. (A) Diagram showing experimental design for tissue-specific WGBS. Ripening-induced DNA methylation differences in skin and pulp were identified by comparing methylomes at ripe relative to immature for each tissue. RI: Ripening-induced methylation differences. (B) DNA methylation profiles of mC, mCG, mCHG, and mCHH contexts surrounding genes and transposable elements for immature and ripe samples in pulp (top) and skin (bottom). Two biological replicates are shown for each tissue and stage. (C) Number of ripening-induced DMRs identified in pulp and skin. The upper plots display the number of hypomethylated DMRs (hypo-DMRs) and lower plots display the number of hypermethylated DMRs (hyper-DMRs).

Grapevine varieties exhibit high levels of heterozygosity, largely due to thousands of years of clonal cultivation that has facilitated the accumulation of extensive genetic variation. We aimed to investigate whether the ripening-induced DNA methylation is conserved across different grapevine varieties. To investigate this conservation, we selected ‘Cabernet Sauvignon’, another globally important cultivar, for a comparative analysis. Additionally, DNA methylation patterns can vary across different tissues within plant organs [4, 21]. However, the tissue-specific DNA methylation patterns in grape berries have not been investigated. We hand-dissected skin (exocarp) and pulp (mesocarp) from ‘Cabernet Sauvignon’ berries and characterized their methylomes at two developmental stages: immature and ripe (Fig. 2A and Fig. S4). All libraries exhibited bisulfite conversion rates exceeding 99.7%, with an average sequencing depth of 28-fold (range 23–35), which provided valid sites (sequencing depth ≥4) covering >81% of genomic cytosines (Table S1). Strong correlation between biological replicates confirmed data reproducibility (Fig. S5).

Investigating ripening-associated methylation dynamics, we observed a notable increase particularly within the CHH context in both skin and pulp tissues (Fig. 2). Remarkably, promoter regions, transposable-element bodies and flanking regions had pronounced CHH hypermethylation across both tissues (Fig. 2B), mirroring the pattern in whole ‘Wink’ berries (Fig. 1D). Methylation changes in other sequence contexts remained minimal (Fig. 2B and C, Fig. S6). These results demonstrate that both c.v. ‘Wink’ and c.v. ‘Cabernet Sauvignon’ grapevine genomes undergo widespread gains in DNA methylation, particularly in CHH methylation, during berry ripening. This occurs in both skin and pulp tissues, underscoring the contribution of DNA methylation to the ripening process.

### Skin and pulp tissues have distinct hypermethylation patterns during ripening

To investigate quantitative changes in DNA methylation during ripening of c.v. ‘Cabernet Sauvignon’, we identified differentially methylated regions (DMRs) by comparing methylomes of skin and pulp tissues at immature and ripe stages. Using the MethylKit R package to account for biological variability [22], we detected 1816 ripening-induced DMRs in skin (RI skin DMRs) and 2626 in pulp (RI pulp DMRs). Hyper-DMRs substantially outnumbered hypo-DMRs, with CHH hyper-DMRs accounting for >91% of hypermethylated regions (CHH hyper-DMRs/total hyper-DMRs in skin and pulp) (Fig. 2C), consistent with the global CHH increase observed during ripening (Fig. 2B and Fig. S6).

Given the overall methylation gain, we analyzed expression of DNA methylation-related genes in both tissues (immature vs ripe) by RNA-seq. Orthologs of *Arabidopsis* DNA demethylases *DEMETER* and *ROS1*, expressed at immature stage in both tissues, were downregulated during ripening (Fig. S7), aligning with the global hypermethylation trend. Conversely, genes encoding CG methylation maintenance machinery (*MET1* and *VIM1* orthologs) were upregulated at ripe. Similar expression changes have also been observed in the Wink berry (Fig. S8). However, their functional relevance remains uncertain, as berries lack active DNA replication or cell division at this stage [23].

**Figure 3:**
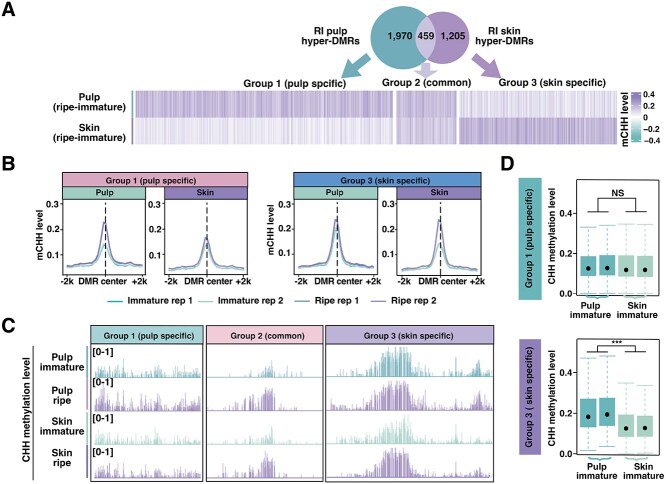
Grape berry tissues exhibit tissue-specific ripening-induced hypermethylation signatures. (A) Venn diagram and heatmaps illustrating CHH methylation difference (ripe–immature) in pulp and skin. Groups 1–3 are ripening-induced (RI) hypermethylated regions specific to pulp, common to both tissues, and specific to skin, respectively. (B) CHH methylation levels (±2 kb from DMR center) in skin and pulp at immature and ripe stages within Group 1 (pulp-specific hyper-DMRs) and Group 3 (skin-specific hyper-DMRs). (C) IGV snapshots showing CHH methylation levels of representative regions of Group 1 (pulp-specific hyper-DMRs), Group 2 (common hyper-DMRs), and Group 3 (skin-specific hyper-DMRs). (D) CHH methylation level at immature stage within Group 1 (pulp-specific hyper-DMRs) and Group 3 (skin-specific hyper-DMRs) regions. Statistical comparisons were performed using a two-sided Wilcoxon rank-sum test. NS indicates no significant difference while asterisks indicate significant difference (***P < 0.001).

To explore tissue-specific methylation regulation, we compared 1661 ripening-induced skin CHH hyper-DMRs and 2423 ripening-induced pulp CHH hyper-DMRs, revealing three distinct groups: 1970 pulp-specific regions (assigned as Group 1), 459 shared between tissues (Group 2), and 1205 skin-specific regions (Group 3) (Fig. 3A and Table S2). We examined mCHH levels of Group 1 and 3 hyper-DMRs in pulp and skin, revealing that Group 1 exhibited elevated mCHH levels in pulp, whereas Group 3 had increased mCHH levels in skin during ripening (Fig. 3B). Representative hypermethylated regions from Groups 1 to 3 are illustrated in Fig. 3C, respectively. These findings indicate that skin and pulp exhibit divergent methylation dynamics despite their shared increase in CHH methylation. Notably, Group 3 hyper-DMRs showed pre-existing methylation accumulation in pulp compared to skin at immature stage, while Group 1 did not display significant intertissue methylation differences (Fig. 3D). These results suggest that ripening-associated hypermethylation patterns are tissue-specific and partially attributable to pre-existing methylation differences between skin and pulp at earlier developmental stages.

### DNA methylation inhibitors delay the ripening of berries grown

**Figure 4:**
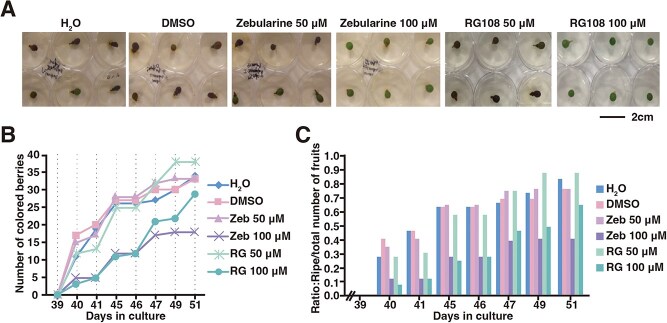
DNA methylation inhibitors limit berry ripening *in vitro*. (A) Representative phenotypes of c.v. ‘Cabernet Sauvignon’ berries after treatment with control (H_2_O, DMSO) or zebularine and RG108 at 50 or 100 μM medium *in vitro*. Berries were collected at 2 weeks postflowering and monitored for color development 40–51 days post-treatment. Scale bar = 2 cm. (B, C) Changes in number of ripened berries (B) and ratio of ripened berries versus the total number of berries grown *in vitro* (C) in control (H_2_O, DMSO) and after zebularine and RG108 treatments at 50 and 100 μM. n=48 berries per condition.

To investigate the functional role of ripening-associated DNA methylation in grape berries, we employed an *in vitro* ripening system [24]. Berries harvested from fruiting cuttings at 2 weeks postflowering were surface-sterilized and cultured in media supplemented with the DNA methyltransferase inhibitors zebularine or RG108, or control solutions (H_2_O or dimethyl sulfoxide (DMSO)). Zebularine (a nucleoside analog of cytidine) and RG108 (a non-nucleoside inhibitor of DNA methyltransferases) were chosen for their distinct methylation inhibition mechanisms, stability, and low toxicity [25–27]. Phenotypes of berries after treatment with control solution (DMSO, H_2_O) or zebularine and RG108 at 50 or 100 μM were shown (Fig. 4A). For control berries (H_2_O or DMSO), the accumulation of pigment was visible at 40 days (~8 weeks after flowering), mirroring *in planta* ripening kinetics (Fig. 4B and C). Control berries showed progressive ripening, reaching 80% ripened berries by Day 51 (Fig. 4B and C). Low-concentration treatments (50 μM zebularine/RG108) caused minimal delays, and berries ripened similarly to controls by Day 51 (Fig. 4B and C). For berries treated with 100 μM zebularine, coloration plateaued by Day 47, with 60% berries remaining unripened (Fig. 4B and C).

For berries treated with 100 μM RG108, ripening was initially suppressed comparably to 100 μM zebularine and was resumed after 47 days, but remained lower than controls, with ~37% unripened berries by Day 51. These results highlight concentration-dependent disruption of ripening, with zebularine exerting stronger inhibitory effects, underscoring the critical role of DNA methylation in grape berry maturation.

### A subset of ripening-related genes possess tissue-specific DNA methylation change and tissue-specific gene expression change during grape ripening

Previous studies showed tissue-specific gene expression in grape berries. To study how tissue-specific DNA methylation dynamics are associated with gene expression, we performed RNA-seq on c.v. ‘Cabernet Sauvignon’ skin and pulp tissues at immature and ripe stages using three biological replicates. Correlation and principal component analysis confirmed reproducibility among replicates (Fig. S9A and B). We first examined the relationship between transcript abundance and methylation levels for active genes (FPKM ≥1) and inactive genes (FPKM <1). Active genes were primarily located in chromosome arms, whereas inactive genes were enriched in pericentromeric regions characterized by high levels of DNA methylation (Fig. S9C). In all tested samples, inactive genes exhibited high promoter and gene body methylation, whereas active genes showed statistically significantly lower methylation (Fig. S9D), consistent with methylation’s role in gene silencing [28, 29].

**Figure 5:**
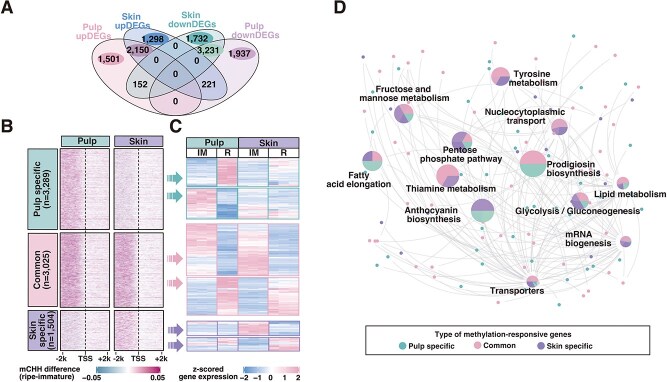
Tissue-specific methylation is associated with genes enriched in metabolite related pathway. (A) Venn diagram exhibiting the overlap of ripening-induced DEGs in the skin and pulp of ‘Cabernet Sauvignon’ berries. (B) Heatmaps showing ripening-induced mCHH differences (±2-kb window around the TSS) in pulp and skin tissues. Three gene categories are shown: genes that are specifically hypermethylated in pulp (pulp-specific), hypermethylated in both tissues (common), and specifically hypermethylated in skin (skin-specific). (C) Heatmaps illustrate gene expression levels in pulp and skin at immature and ripe stage for methylation-responsive genes, which exhibit significant DNA methylation and transcriptional changes between tissues. Data are presented as *z*-scored gene expression values. (D) Network diagram highlighting significantly enriched metabolic pathways associated with methylation-responsive genes. Node color indicates gene category (common, pulp-specific, or skin-specific). Edges connect genes to enriched KEGG pathways. Pie charts within the nodes represent the proportion of each gene category contributing to the pathway enrichment.

Secondly, our analysis of differentially expressed genes (DEGs) between these developmental stages revealed substantial transcriptome changes during ripening. In pulp, 5389 genes were downregulated and 3803 upregulated; in skin, 5115 genes were downregulated and 3669 upregulated. Considerable overlap was observed among DEG profiles between the two tissues, with 3231 genes downregulated and 2150 genes upregulated in both skin and pulp (Fig. 5A). These findings indicate that grape berries underwent both tissue-specific gene regulation and tissue-shared gene regulation during ripening. To further investigate the correlation of tissue-specific methylation and gene expression during ripening, we categorized genes based on their methylation changes in pulp and skin tissues, resulting in three categories: 3289 genes that are specifically hypermethylated in pulp (pulp-specific hyper-mCHH genes), 3025 genes that are hypermethylated in both tissues (common hyper-mCHH genes), and 1504 genes that are specifically hypermethylated in skin (skin-specific hyper-mCHH genes) (Fig. 5B). Among pulp-specific hyper-mCHH genes, we identified 301 genes (144 up- and 157 downregulated genes) that are differentially expressed only in pulp. Among skin-specific hyper-mCHH genes, we identified 140 genes (66 up- and 74 downregulated genes) differentially expressed only in skin, suggesting that the tissue-specific expression of these genes may involve tissue-specific hypermethylation during ripening. Among the common hyper-mCHH genes, 442 genes (187 up- and 255 downregulated genes) are differentially expressed in both tissues (Fig. 5C and Table S3). To explore the functional implications of methylation-responsive genes, we performed KEGG enrichment analysis of these three groups, revealing association with grape ripening-related metabolic pathways, such as anthocyanin biosynthesis, fructose and mannose metabolism, biosynthesis of various secondary metabolites, glycolysis/gluconeogenesis, and tyrosine metabolism pathway (Fig. 5D and Table S4).

In summary, our data identified a cluster of ripening-related genes whose tissue-specific gene expression pattern is associated with their tissue-specific DNA methylation change, and we also found a group of genes whose expression and DNA methylation levels are similarly regulated in both tissues.

### Tissue-specific metabolic profiles are associated with tissue-specific methylation-responsive genes

**Figure 6:**
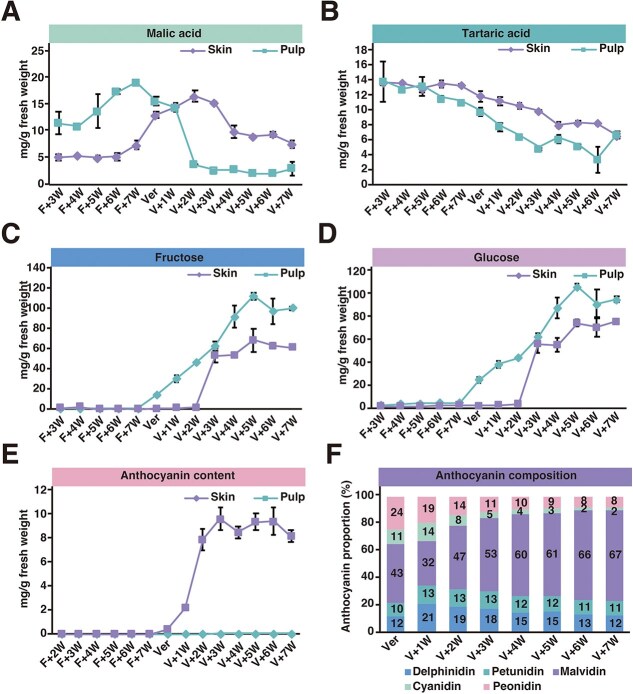
Changes in signature metabolite profiles during ‘Cabernet Sauvignon’ grape berry ripening. The contents of (A) malic acid, (B) tartaric acid, (C) fructose, (D) glucose, and (E) total anthocyanins were measured in both skin and pulp at 15 successive ripening stages. (F) The composition of individual anthocyanins was analyzed in the skin after veraison. Data are presented as mean ± SD (*n* = 48).

**Figure 7:**
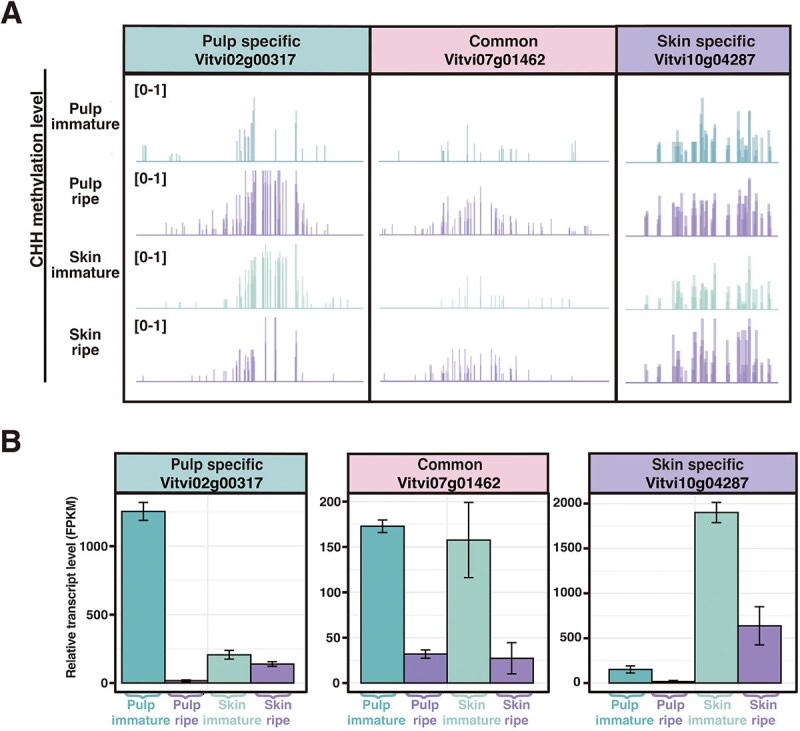
Tissue-specific metabolic profiles are associated with tissue-specific methylation-responsive genes. (A) IGV snapshots illustrating methylation (mCHH) for representative methylation-responsive genes in pulp and skin at immature and ripe stages. Three genes are shown: *Vitvi02g00317* (pulp-specific), *Vitvi07g01462* (common), and *Vitvi10g04287* (skin-specific). (B) Relative transcript levels of *Vitvi02g00317* (pulp-specific), *Vitvi07g01462* (common), and *Vitvi10g04287* (skin-specific) in pulp and skin at immature and ripe stage.

The KEGG analysis showed that the methylation-responsive genes are enriched in many metabolism-related pathways. We hypothesized that the expression pattern of these methylation-responsive genes might be associated with the accumulation of metabolites during ripening. We investigated the accumulation kinetics of specialized metabolites that are hallmarks of grape ripening. Skin and pulp tissues were dissected from c.v. ‘Cabernet Sauvignon’ berries at 15 successive developmental stages from the first week after flowering to the seventh week after veraison and their metabolite profiles were characterized to quantify organic acids, soluble sugars, and anthocyanins (Fig. 6). Malic acid declined after veraison in both tissues with a faster decline in pulp, while tartaric acid displayed a similar gradual decline in both tissues (Fig. 6A). However, analysis of methylation-responsive genes revealed no significant enrichment of genes associated with the acid metabolic pathway (Fig. 5D). Fructose and glucose exhibited similar accumulation kinetics in both tissues with a gradual increase over time (Fig. 6C and D). Within the genes enriched in the ‘fructose and mannose metabolism’ KEGG pathway (Fig. 5D), we found that the promoter region of *Vitvi07g01462* was hypermethylated in both the skin and pulp, accompanied by downregulation of its expression in both tissues (Fig. 7). This gene encodes an ATP-dependent 6-phosphofructokinase (PFK) that catalyzes the phosphorylation of fructose 6-phosphate (F6P) to fructose 1,6-bisphosphate (FBP). This is considered a critical step in directing carbon flux into glycolysis, and this enzyme negatively regulates hexose levels in plants [30]. The methylation and expression pattern of *Vitvi07g01462* is consistent with the observed fructose accumulation in both skin and pulp during the ripening process (Fig. 7).

The most contrasting accumulation profiles were observed for anthocyanins. As expected, anthocyanins were undetectable in the pulp at all stages. In contrast, skin anthocyanin content increased sharply from veraison, rapidly reaching a maximum concentration of 9.7 ± 0.94 mg/g fresh weight at V + 3 W, with minimal subsequent fluctuation (Fig. 6E). Further analysis of the anthocyanin composition revealed 22 distinct anthocyanins and their derivatives, with a progressive increase in the proportion of trihydroxylated compounds (delphinidin, petunidin, and malvidin derivatives) from 65% to 90% of the total anthocyanin content over the 7 weeks following veraison. Specifically, the proportion of delphinidin increased significantly, while petunidin and malvidin derivatives also contributed to this rise (Fig. 6F). In apple and pear, methylation of *MYB* genes correlates with anthocyanin deposition [31–34]. In our data, we did not find methylation change in grape MYB orthologs in skin or pulp (Fig. S10). In addition, we did not observe methylation change for genes encoding enzymes directly involved in anthocyanin biosynthesis during ripening (Fig. S10). However, within the genes enriched in tyrosine metabolism pathway (Fig. 5D), we identified *Vitvi10g04287*, a gene-encoding polyphenol oxidase (PPO) that is widely involved in the oxidative degradation of anthocyanin [35]. PPO orthologs in sweet potato, cranberry, and litchi actively regulate anthocyanin stability. Sweet potato PPO selectively binds acylated anthocyanins [36], cranberry PPO oxidizes cyanidin glycosides [35], and litchi PPO collaborates with peroxidase to degrade anthocyanidins after they are hydrolyzed by anthocyanase, collectively promoting postbiosynthetic pigment loss [37]. We observed skin-specific hypermethylation in the promoter region of *Vitvi10g04287*, concurrent with skin-specific expression downregulation during ripening. This aligns with skin-specific anthocyanin accumulation. In addition, we noted *Vitvi02g00317*, which is predicted to encode a GLABRA 3 (GL3) TF belonging to the bHLH subgroup IIIf-2 subfamily. In *Arabidopsis thaliana*, GL3 positively regulates anthocyanin biosynthesis [38]. Here, the *Vitvi02g00317* promoter region showed pulp-specific hypermethylation during ripening, concurrent with pulp-specific downregulation of gene expression (Fig. 7). This observation is consistent with the absence of anthocyanin accumulation in the pulp. These results suggested that DNA methylation might influence expression of genes involved in anthocyanin metabolism, although it didn’t directly influence genes encoding enzymes in anthocyanin biosynthesis.

Beyond traditional biochemical and genetic approaches, structure-guided analyses have become instrumental in dissecting protein function. Leveraging this strategy, we performed molecular docking simulations to probe the interaction between the *Vitvi10g04287*-encoded PPO and cyanidin-3-arabinoside, a key anthocyanin pigment found in various berries, including grapes, thereby illuminating its potential role in anthocyanin metabolism [39]. Using AlphaFold3, we predicted the 3D structure of the PPO protein and validated its reliability by demonstrating high structural similarity to the crystal structure of *V. vinifera* PPO (PDB: 2P3X) [40]. Superimposition of the predicted model and the crystal structure yielded a root mean square deviation (RMSD) of just 0.460 Å, confirming the high accuracy of our model (Fig. S11A). Molecular docking simulations revealed that PPO forms stable interactions with cyanidin-3-arabinoside, with a mean binding energy of −7.5 kcal/mol (range: −8.0 to −7.1 kcal/mol), significantly stronger than the control 3,4-dihydroxyphenylacetic acid (−5.7 kcal/mol) (Fig. S11B). Structural analysis further identified hydrogen bonds between PPO and cyanidin-3-arabinoside at residues Ile3, Ser11, and Ala94, which likely contribute to the specificity and stability of their interaction (Fig. S11C and D). These findings provide computational evidence that *Vitvi10g04287* encodes a functional PPO involved in anthocyanin metabolism.

## Discussion

DNA methylation is an essential mechanism controlling fruit ripening in both climacteric and nonclimacteric fleshy fruits, although the underlying mechanisms differ between species. For example, in tomato and strawberry fruit, demethylation is an absolute requirement for ripening to occur. In tomato, demethylation is mediated by a rise in expression of *SlDML2*, which encodes a DNA demethylase [2, 3], while in strawberry, reduced DNA methylation is achieved by downregulation of the RdDM pathway [11]. In contrast, orange fruit show an increase in methylation associated with fruit ripening [12]. This indicates that though control of DNA methylation is clearly involved in fruit ripening, its role and the underlying mechanisms differ across taxa [41]. Here, we sought to investigate the patterns and roles of DNA methylation during grape berry ripening. The patterns observed in grape berries are similar to those described in orange fruits [12]. We constructed single-base-resolution DNA methylation profiles of grape berries during ripening and saw that berries undergo a genome-wide DNA methylation increase, particularly in the CHH context. This situation is in sharp contrast to strawberry [11] and tomato [2]. In the case of grapes, increased DNA methylation is associated with a repression of demethylase genes (Fig. S7) akin to orange [12].

Most studies of epigenetic regulation in fleshy fruits analyze whole organs rather than distinct tissues, with tomato tissue-specific methylation analysis a rare exception [21]. In contrast, our analysis of grape-berry pulp and skin revealed globally similar methylation trends during ripening, contrasting with tomato fruit tissues wherein pericarp methylation declines while locular tissue methylation remains stable [21]. This underscores fundamental differences in methylation regulation between these species. Notably, grape berry tissues exhibited similar methylation dynamics across sequence contexts (CG, CHG, CHH) and genomic features. However, tissue-specific methylation differences emerged at localized genomic regions, as evidenced by thousands of differentially methylated regions between pulp and skin at both developmental stages studied (Fig. 3). These findings suggest that while global methylation dynamics are conserved across tissues, localized epigenetic reprogramming instead targets distinct loci—likely reflecting tissue-specific functional requirements.

Fruit ripening involves coordinated biochemical and physiological transitions, including chlorophyll degradation, anthocyanin accumulation (leading to color changes from green to red), sugar accumulation, shifts in acid content, and biosynthesis of aromatic compounds. While some metabolic changes are shared across fruit tissues, others are tissue-specific. we measured ripening-induced changes in five key metabolites—malic and tartaric acid, fructose, glucose, and anthocyanins. Among the five metabolites, anthocyanins accumulated exclusively in skin post-veraison, remaining undetectable in pulp (Fig. 6). The methylation inhibitor assay demonstrated that DNA hypermethylation was required for coloration (Fig. 4). A combined analysis of methylome and transcriptome showed that ripening-related DNA hypermethylation didn’t directly regulate anthocyanin biosynthesis genes (e.g. CHS, DFR). However, tissue-specific methylation patterns may correlate with anthocyanin degradation. For instance, skin-specific hypermethylation of the polyphenol oxidase gene *Vitvi10g04287* coincided with its transcriptional repression and the concurrent accumulation of anthocyanin in the skin. This gene, involved in anthocyanin degradation, may be suppressed to prevent pigment breakdown. The transcriptional activation of anthocyanin structural genes is orchestrated by an evolutionarily conserved regulatory module known as the MYB–bHLH–WDR (MBW) complex. This heterotrimeric complex is formed by the assembly of three distinct proteins: an R2R3-MYB TF, a basic helix–loop–helix (bHLH) TF, and a WD-repeat protein [42–44]. Within this regulatory unit, the R2R3-MYB factors are considered the key activating components, and their pivotal role has been extensively characterized in diverse plant species, such as petunia (*Petunia hybrida*) [43], *A. thaliana* [44, 45], apple [46–48], and pear [49, 50]. Although DNA methylation was involved in regulation of MYB in apple [31, 32] and pear during ripening [33, 34], we didn’t observe ripening-related DNA hypermethylation in R2R3-MYB genes in grape berries (Fig. S10). However, we found that a bHLH TF GL3 (*Vitvi02g00317*), a part of the MBW complex, underwent pulp-specific hypermethylation during ripening, and this hypermethylation is associated with suppressed gene expression, aligning with the absence of anthocyanins in pulp. These results imply that methylation modulates genes in ancillary pathways (e.g. tyrosine metabolism) rather than directly targeting biosynthetic enzymes. Thus, DNA methylation likely fine tunes anthocyanin metabolism by controlling genes involved in pigment stabilization or transcriptional regulation, underscoring its role as a contextual epigenetic switch during ripening.

## Conclusions

This study reveals that DNA methylation plays a critical and conserved role in grape berry ripening, with a genome-wide increase in methylation, particularly in the CHH context. While global methylation dynamics are shared across skin and pulp tissues, localized epigenetic reprogramming targets distinct genomic regions, reflecting tissue-specific functional requirements during ripening. These findings highlight DNA methylation as a key regulator of ripening-associated processes, including anthocyanin accumulation and sugar metabolism, providing new insights into the epigenetic control of fruit development in nonclimacteric species.

## Materials and methods

### Plant materials

Grapevine (*V. vinifera*) plants were grown at the fruit experimental station located in Nanjing, China (N 32°02′12.77″, E 118°37′33.25″). Berries from 3-year-old c.v. ‘Wink’ plants were sampled at the immature (38 DPA), veraison (59 DPA), and ripe (87 DPA) stages throughout the growing season in 2015. The WGBS analysis of the ‘Wink’ cultivar was carried out on whole, nondissected berries after seed removal. For tissue-specific methylome sequencing, all experiments were carried out using c.v. ‘Cabernet Sauvignon’. ‘Cabernet Sauvignon’ grape berries were collected from the VitAdapt experimental block [51] at INRAE research station located at Villenave d’Ornon, France (44°47′23.83″ N, 0°34′39.3″ W). Grapevines were planted in 2009 and berries were harvested at 15 successive developmental stages from the first week after flowering to the seventh week after veraison from May to October. As the development of grape berries is not synchronized in one cluster, berries were labeled at two different times: flowering and veraison phase. Individual berries were labeled at the ripening phase when berries started to turn red. Fruits at immature (6 weeks after flowering) and ripe (3 weeks after veraison) stages were used in DNA methylation analysis.

In total, 1509 individual berries were labeled at the veraison stage, and labeling and harvest of berries were performed at the same hour every day (9–10 a.m.). Before veraison, berries were collected based on the flowering time and the size of berry. During the sampling process, various parameters related to berry development and ripening were recorded including size and diameter. Berries were counted and total weight was measured to determine the mean berry weight. Seven berries were randomly selected at each stage, squeezed out to obtain three drops of juice from each berry, which was analyzed with a hand refractometer to measure soluble solids °Bx (degrees Brix). Extracted juice was analyzed using a pH meter (Hanna Instruments, UK). Berry samples were flash-frozen in liquid nitrogen right after collection and further processed to separate skin from flesh, with seeds being discarded (except the first two stages). These separated tissues were subsequently ground into a fine powder under liquid nitrogen conditions using a Retsch MM200 ball grinder and stored at −80°C pending analysis.

### WGBS library preparation, sequencing, and bioinformatic analysis

Genomic DNA from grape berries was isolated using Qiagen DNeasy Kits (Maxi or Mini) and prepared for WGBS with the NEBNext II DNA Library Prep Kit. Sequencing was carried out on an Illumina HiSeq2500 instrument.

Raw paired-end sequencing reads were quality-filtered and adapter-trimmed using Fastp [52] (quality score <20). Cleaned reads were then aligned to the *V. vinifera* PN40024 (v4) reference genome [53] using Bismark v0.23.0 [54], allowing for one mismatch during mapping. Duplicate reads were removed via deduplicate_bismark. Methylation levels for each cytosine were extracted via bismark_methylation_extractor, retaining only sites with read depth ≥4 for further analysis. The methylation level of chloroplast DNA was used to assess the bisulfite conversion efficiency. Data visualization was performed using the Integrative Genomics Viewer (IGV) [55].

The genome was partitioned into 1-kb bins to assess sample-wide methylation patterns and reproducibility through Pearson correlation analysis. The R package MethylKit [22] was employed to identify DMCs and DMRs. The built-in logistic regression method in MethylKit was employed to calculate *P*-values. DMCs were called based on context-specific methylation differences (CG: >20%; CHG: >10%; CHH: >5%) and a *P*-value of <0.05. DMRs were identified using a 200-bp sliding window (50-bp step) and refined by merging proximal candidate regions (within 100 bp) and filtering for those containing at least 10 DMCs.

To identify methylation-responsive genes, we divided the 2-kb promoter region of each gene into 20 bins of 100 bp each. We then calculated the mCHH difference for each bin. Bins with an mCHH value >0.05 were defined as differentially methylated bins. A gene was considered methylation-responsive if it had two or more differentially methylated bins. In Table S3, we’ve listed the number of differentially methylated bins for each gene and used the average DNA methylation difference of these bins as the mCHH difference for the gene.

### Transcriptome library preparation, sequencing, and bioinformatic analysis

Total RNA was isolated from fruit skin and pulp tissues using TRIzol reagent (Ambion). For each sample, 1 μg of total RNA served as the template for first-strand cDNA synthesis, which was performed with oligo(dT) primers using the qScript cDNA SuperMix kit (Quanta). The subsequent construction of RNA-seq libraries and paired-end sequencing were carried out on an Illumina HiSeq2500 platform.

Bioinformatic analysis commenced with raw paired-end sequencing reads using Fastp [52] to remove adapter sequences and low-quality data. The cleaned, high-quality reads were then aligned to *V. vinifera* PN40024(v4) [53] reference genome utilizing the STAR [56] aligner with its default settings. Post-alignment, the number of reads uniquely mapping to each transcript was quantified via the ‘htseq-count’ function within the HTSeq [57] Python library. The resulting matrix of raw counts was imported into the R environment, where the R package edgeR [58] was employed to identify DEGs.

To functionally characterize the grape transcriptome, all protein-coding sequences were annotated using eggNOG-mapper [59, 60], which assigns function based on orthology. Finally, Gene Ontology (GO) and KEGG pathway enrichment analysis for sets of methylation-responsive genes were conducted using TBtools-II [61].

### *In vitro* culture and chemical treatment of grape berries

An *in vitro* culture system was established using berries from *V. vinifera* L*.* c.v. ‘Cabernet Sauvignon’ [62] propagated under greenhouse environments. Berries were harvested with their pedicels attached 2 weeks post-anthesis. A rigorous surface sterilization protocol was employed as described in [24] with minor modifications. Briefly, berries were first washed for 15 min under running tap water, followed by sequential immersion in 70% (v/v) ethanol and 2% (v/v) NaClO, concluding with four rinses in sterile water. To prevent sieve tube occlusion by callose, the pedicel base was submerged in a 20 mM EDTA solution, and the pedicel was recut within this solution. Prepared berries were individually cultivated in six-well plates (Dutscher, 353 046) containing 5 ml of culture medium per well. The cultures were maintained in a growth chamber under controlled conditions, with a constant temperature of 26 ± 0.5°C, a 16/8 h (day/night) photoperiod, and an irradiance of ~50 μmol photons m^−2^ s^−1^. Contamination was checked and recorded every day.

To analyze the role of DNA methylation in the ripening process, a specialized ripening medium was utilized. The basal medium was Murashige and Skoog (MS), supplemented to induce ripening with 60 g/l glucose (Duchefa, S0809), 0.25 g/l N-Z-AmineA (Sigma, C7290), and a vitamin cocktail (100 mg/l myo-inositol, 1.0 mg/l nicotinic acid, 1.0 mg/l pantothenic acid, 0.01 mg/l biotin, 1.0 mg/l pyridoxine HCl, and 1.0 mg/l thiamine HCl). The medium pH was adjusted to 5.8 using 0.5 M KOH and solidified with 9 g/l agar (Kalys, HP 696) before being autoclaved.

For the experiment, surface-sterilized berries were cultured on this MS-based ripening medium, which was supplemented with either DNA methyltransferase inhibitors or their respective controls. The treatment groups consisted of zebularine or RG108 (each at 50 and 100 μM), both dissolved in DMSO. Control groups included medium with DMSO alone and a basal control with sterile water. During the *in vitro* culture, contamination rates were 12.5%, 8.3%, 8.3%, 6.25%, 8.3%, and 4.2% in MS supplemented with water, DMSO, 50 μM zebularine, 100 μM zebularine, 50 μM RG108, and 100 μM RG108, respectively.

### Extraction and quantification of soluble sugars, organic acids, and anthocyanins

Soluble sugars and organic acids were extracted from 500 mg of fresh, powdered pulp and skin tissue. The extraction was performed at 80°C by sequential maceration in 2 ml of 80% (v/v) ethanol, 50% (v/v) ethanol, and finally ultrapure water. The combined supernatants from these extractions were evaporated to dryness using a Speed-Vac concentrator (Savant Instruments, Inc.). The resulting residue was reconstituted in 1 ml of ultrapure water (Millipore) and stored at −20°C prior to analysis. Following the procedure described in [63], glucose and fructose concentrations were determined enzymatically using an Elx800UV automated microplate reader (Biotek Instruments Inc.). Tartaric and malic acids were quantified using a TRAACS 800 autoanalyzer (Bran & Luebbe), as described in [64].

For anthocyanin analysis, 400 mg of fresh tissue powder was first lyophilized (freeze-dried) for 72 h. The dried powder was then subjected to extraction with 500 μl of an acidified methanol solution (0.1% HCl in methanol). Prior to analysis, the extracts were clarified by passing them through a 0.2-μm syringe filter. The quantification of individual anthocyanins was performed by High-Performance Liquid Chromatography (HPLC) according to the method described in [65], for which a 3-μl aliquot of the filtered extract was injected for analysis. Anthocyanins were quantified by integrating the absorbance signal at 520 nm. Concentrations were calculated relative to a standard curve generated with a commercial malvidin 3-glucoside standard (Extrasynthese) and were expressed as malvidin 3-glucoside equivalents.

## Supplementary Material

Web_Material_uhaf238

## Data Availability

The raw sequencing data generated during this study are publicly available in the Gene Expression Omnibus (GEO) database under accession numbers GSE279794 and GSE279795 (https://www.ncbi.nlm.nih.gov/geo/). The DNase-seq data reanalyzed for this study can be found in the Sequence Read Archive (SRA) under accession number SRR5405286.
