# Supplementary material for: The grape berry methylome reveals tissue-specific features associated with metabolism in ripening
Source: Hortic Res. 2025 Sep 17;12(12):uhaf238. doi: 10.1093/hr/uhaf238 (PMC12682070; doi:10.1093/hr/uhaf238)
Supplement: Web_Material_uhaf238 [file web_material_uhaf238.zip › Supplementary figures_revised_clean.docx]

Supplementary information

# Supplementary figures

[Figure S1. Pearson Correlation Coefficients between Two Biological Replicates across Different Maturation Stages and DNA Sequence Contexts in ‘Wink’ grape berry methylome 2](#_Toc203981785)

[Figure S2. Density and DNA methylation levels of CG (A-B), CHG (C-D), and CHH (E-F) trinucleotide sequence contexts in ‘Wink’ grape berry methylome. 3](#_Toc203981786)

[Figure S3. Characterization of ripening-induced DNA hypermethylation in ‘Wink’ grape berries. 4](#_Toc203981787)

[Figure S4. Pictures of ‘Cabernet Sauvignon’ grape berries at immature (F+6W) and ripe (V+3W) stage. 5](#_Toc203981788)

[Figure S5. Replicate correlation analysis of genome-wide DNA methylation levels in 'Cabernet Sauvignon' grape skin and pulp at immature and ripe stages. 6](#_Toc203981789)

[Figure S6. DNA methylation differences (ripe - immature) in the gene body and flanking 2k regions of ‘Cabernet Sauvignon’ grape berry skin and pulp. 7](#_Toc203981790)

[Figure S7. Relative transcript levels of genes involved in DNA methylation pathways in the skin and pulp of 'Cabernet Sauvignon' at immature and ripe stages. 8](#_Toc203981791)

[Figure S8. Relative transcript levels of genes involved in DNA methylation pathways in the whole berry of 'Wink' at immature and ripe stages. 9](#_Toc203981792)

[Figure S9. Association between DNA methylation and gene expression during grape fruit ripening. 10](#_Toc203981793)

[Figure S10. Association between DNA methylation and gene expression in the skin and pulp of 'Cabernet Sauvignon' at immature and ripe stages for genes involved in anthocyanin biosynthesis. 11](#_Toc203981794)

[Figure S11. Molecular docking analysis of the interaction between Vitvi10g04287-encoded polyphenol oxidase (PPO) and cyanidin-3-arabinoside. 12](#_Toc203981795)


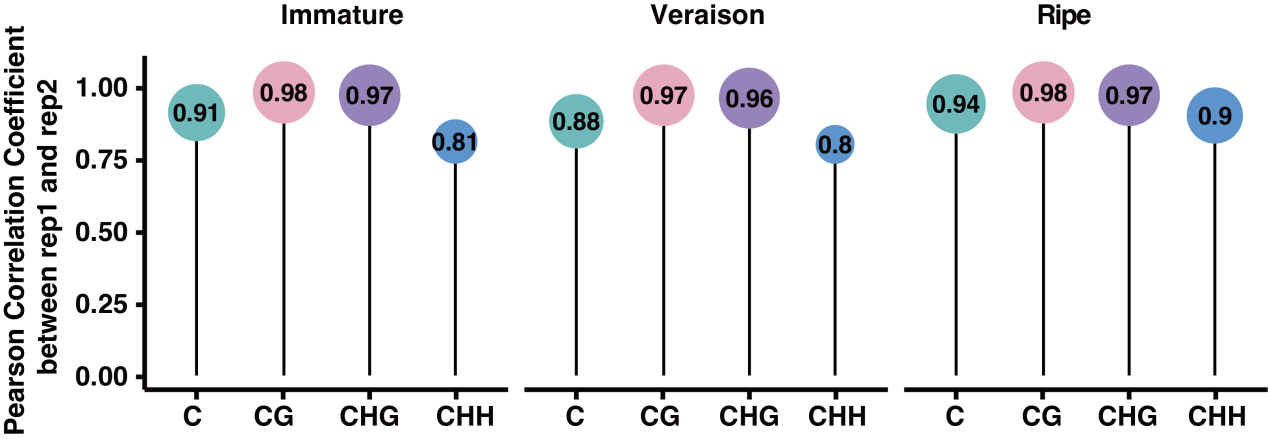


Figure S1. Pearson Correlation Coefficients between Two Biological Replicates across Different Maturation Stages and DNA Sequence Contexts in ‘Wink’ grape berry methylome


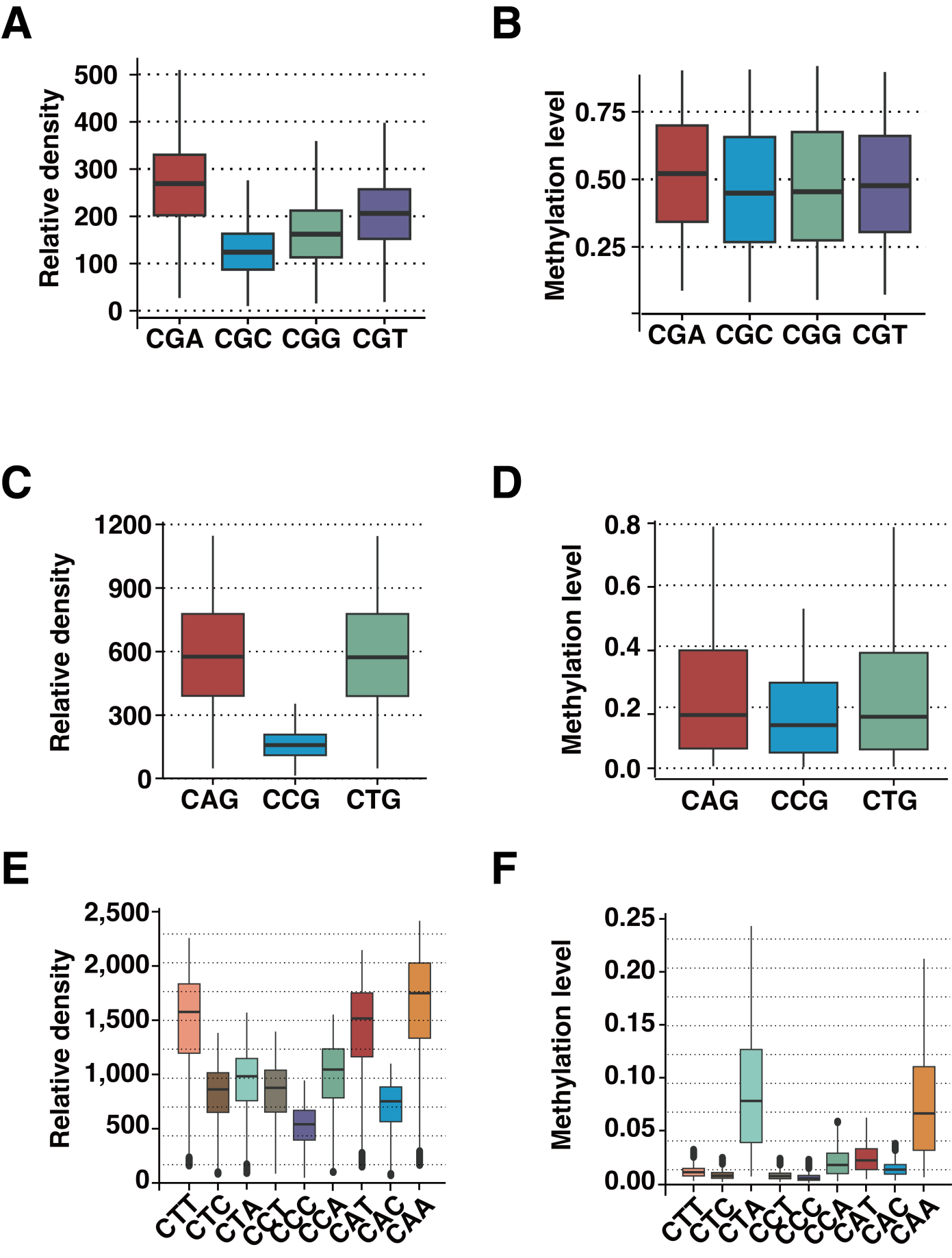


Figure S2. Density and DNA methylation levels of CG (A-B), CHG (C-D), and CHH (E-F) trinucleotide sequence contexts in ‘Wink’ grape berry methylome.


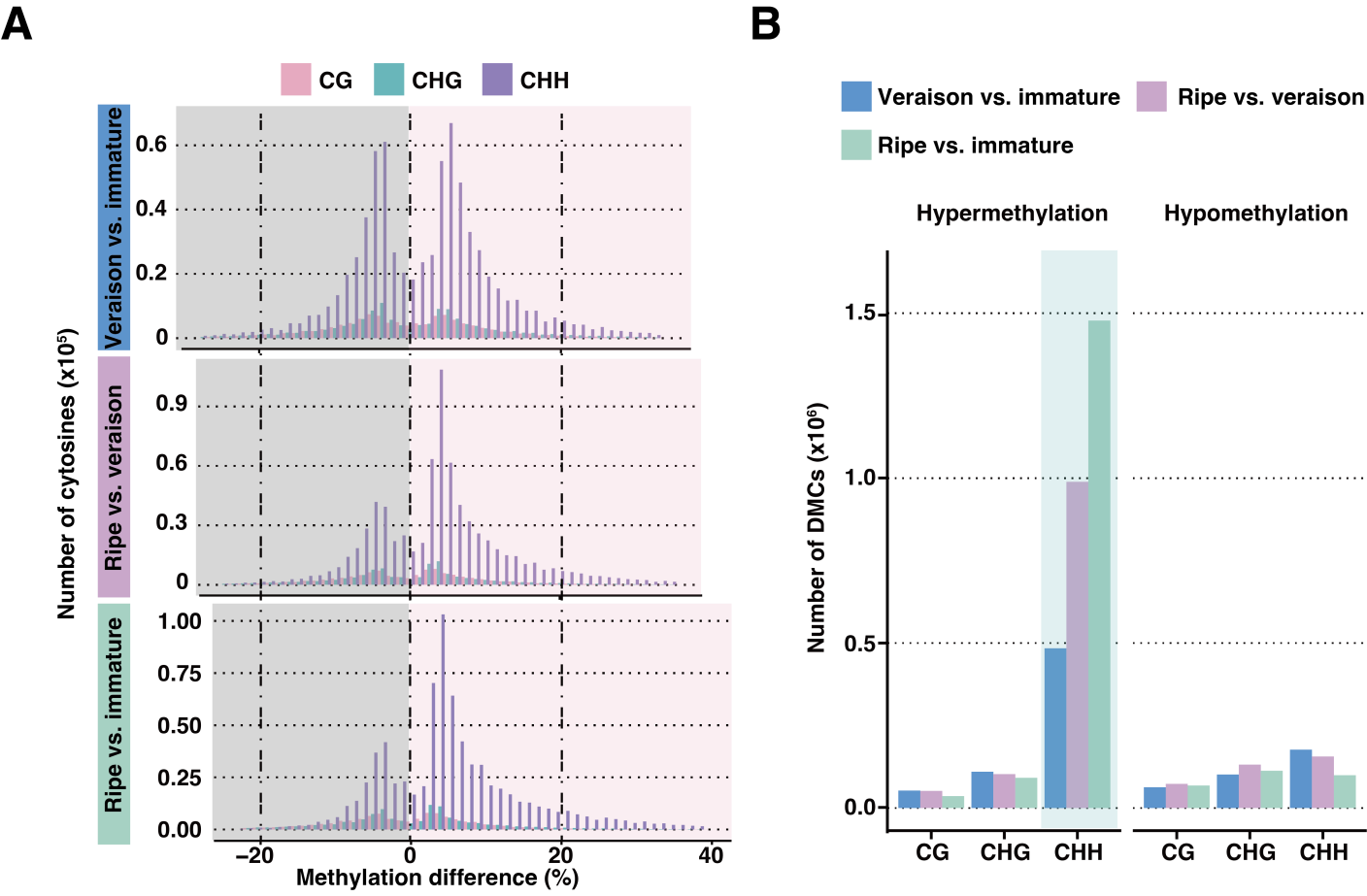


Figure S3. Characterization of ripening-induced DNA hypermethylation in ‘Wink’ grape berries.

(A) Distribution of methylation differences in CG, CHG, and CHH contexts during ripening: veraison vs. immature, ripe vs. veraison, and ripe vs. immature. Cytosines with no methylation difference were excluded.

(B) Number of ripening-induced differentially methylated cytosines (DMCs) identified in pairwise comparisons among immature, veraison, and ripe stages.


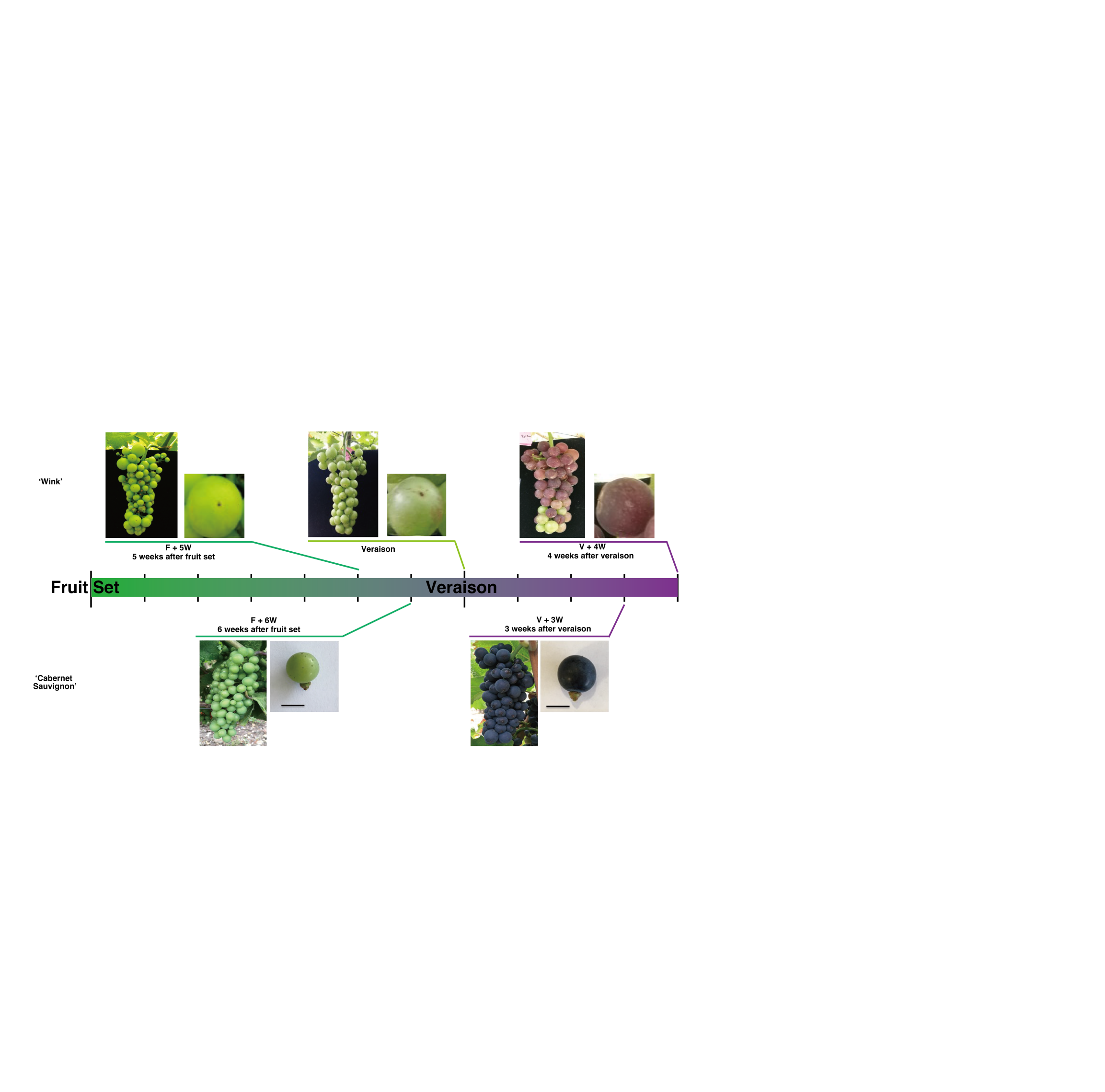


Figure S4. Pictures of ‘Cabernet Sauvignon’ grape berries at immature (F+6W) and ripe (V+3W) stage.


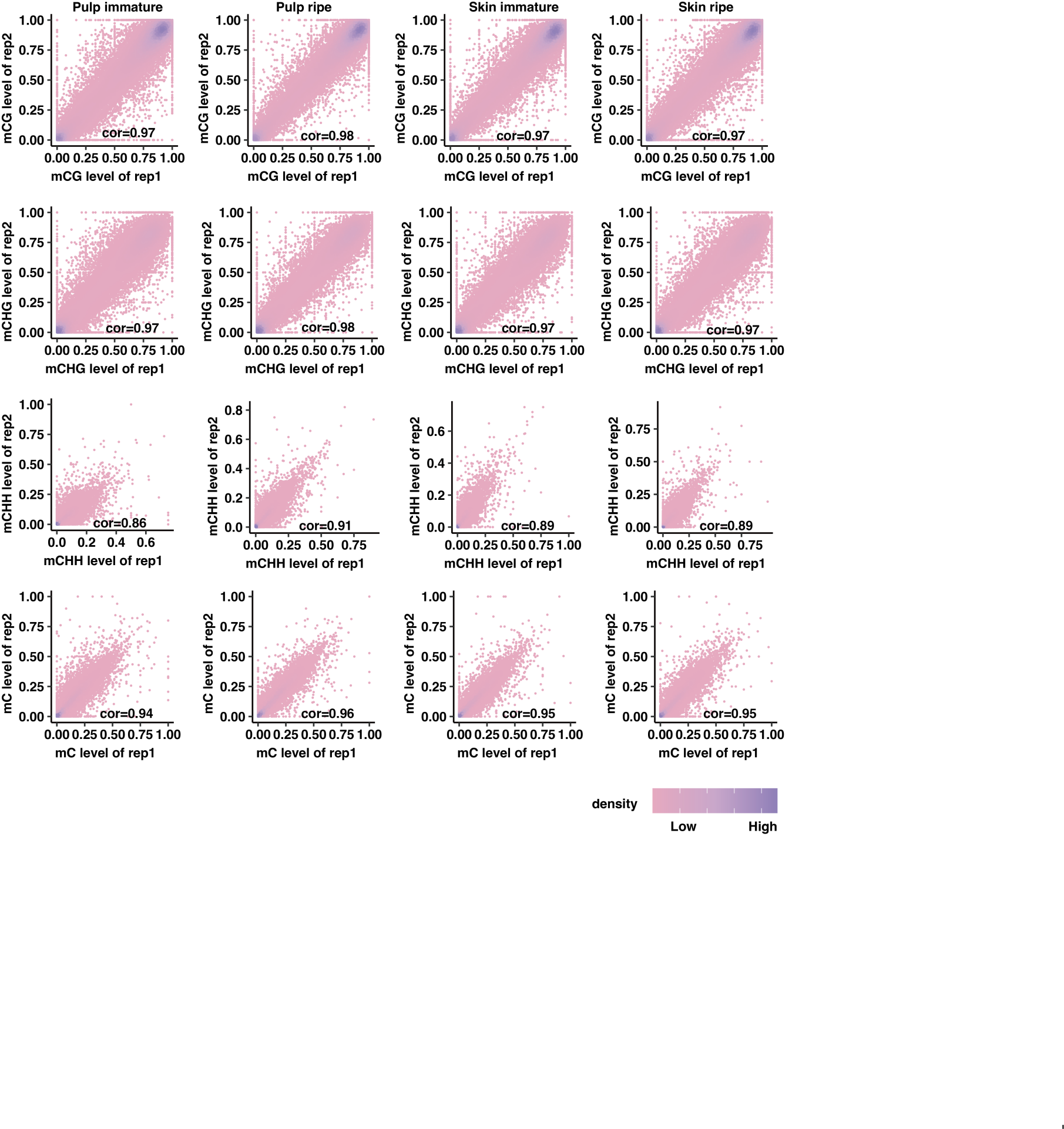


Figure S5. Replicate correlation analysis of genome-wide DNA methylation levels in 'Cabernet Sauvignon' grape skin and pulp at immature and ripe stages.

Methylation levels were calculated for CG, CHG, CHH contexts, and total cytosines. Pearson correlation coefficients for methylation levels in each context were computed to evaluate the reproducibility between replicates for each methylation context. Each row represents the correlation analysis for a specific context (CG, CHG, CHH, or total cytosines).


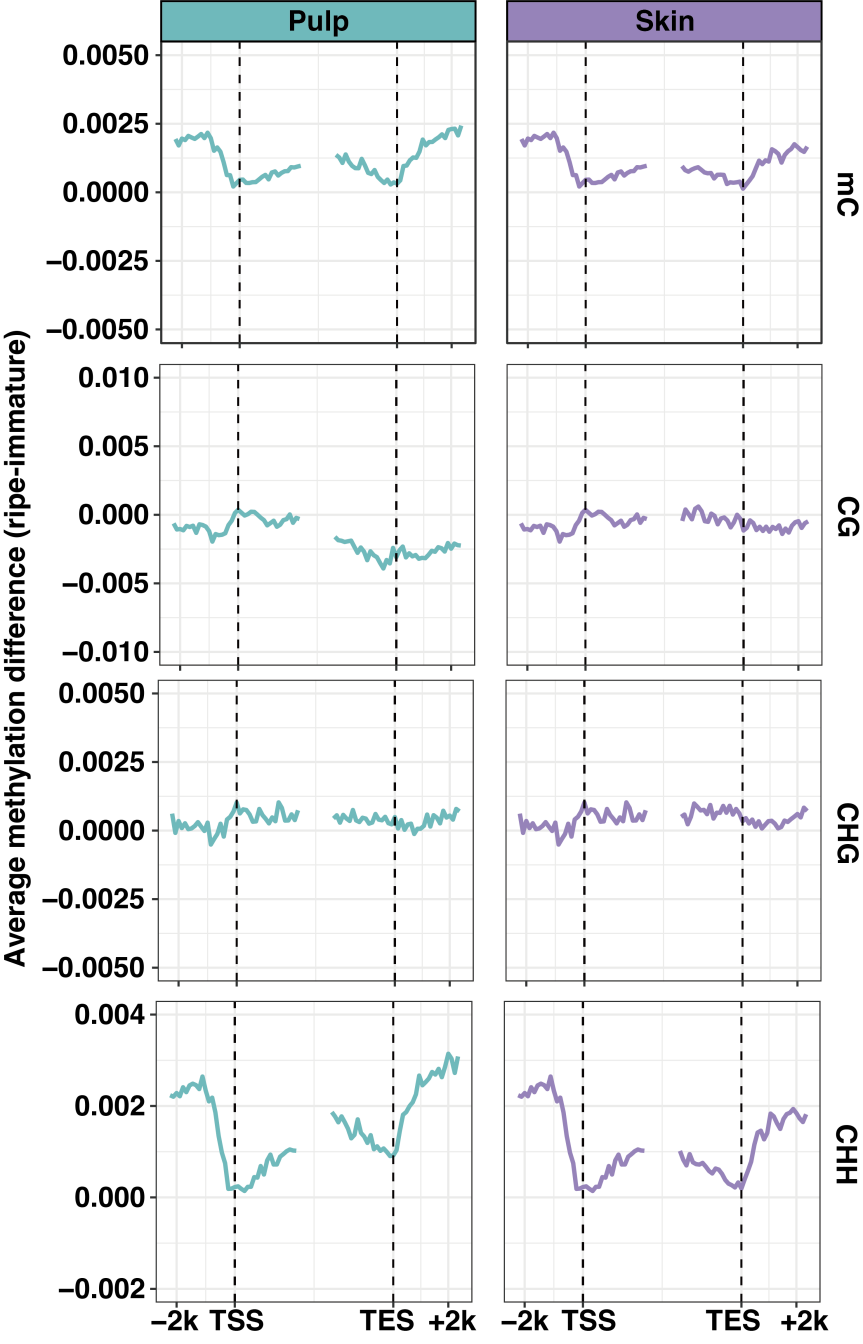


Figure S6. DNA methylation differences (ripe - immature) in the gene body and flanking 2k regions of ‘Cabernet Sauvignon’ grape berry skin and pulp.


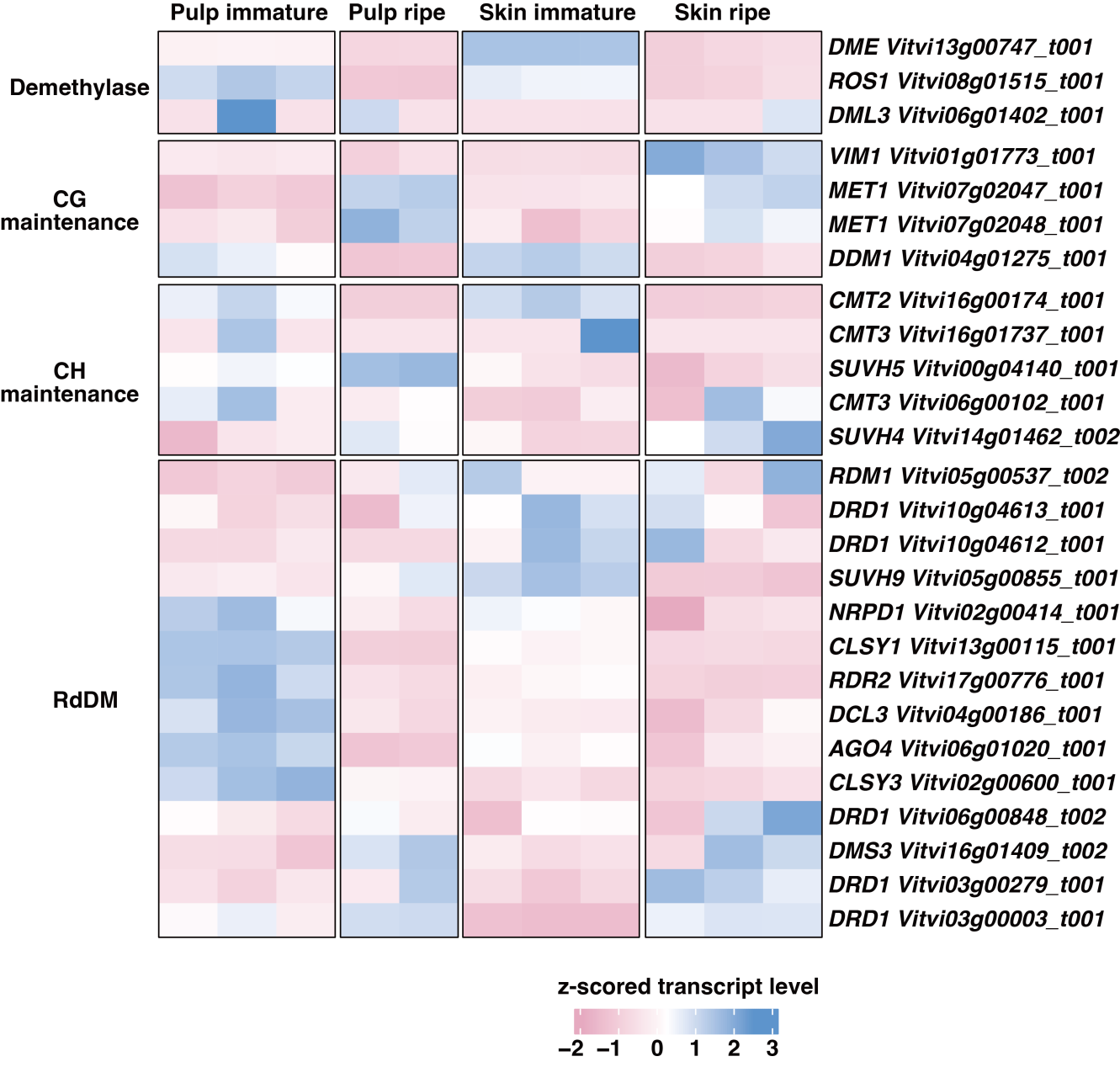


Figure S7. Relative transcript levels of genes involved in DNA methylation pathways in the skin and pulp of 'Cabernet Sauvignon' at immature and ripe stages.

The heatmap displays genes associated with DNA demethylation, DNA methylation maintenance, and *de novo* DNA methylation processes.


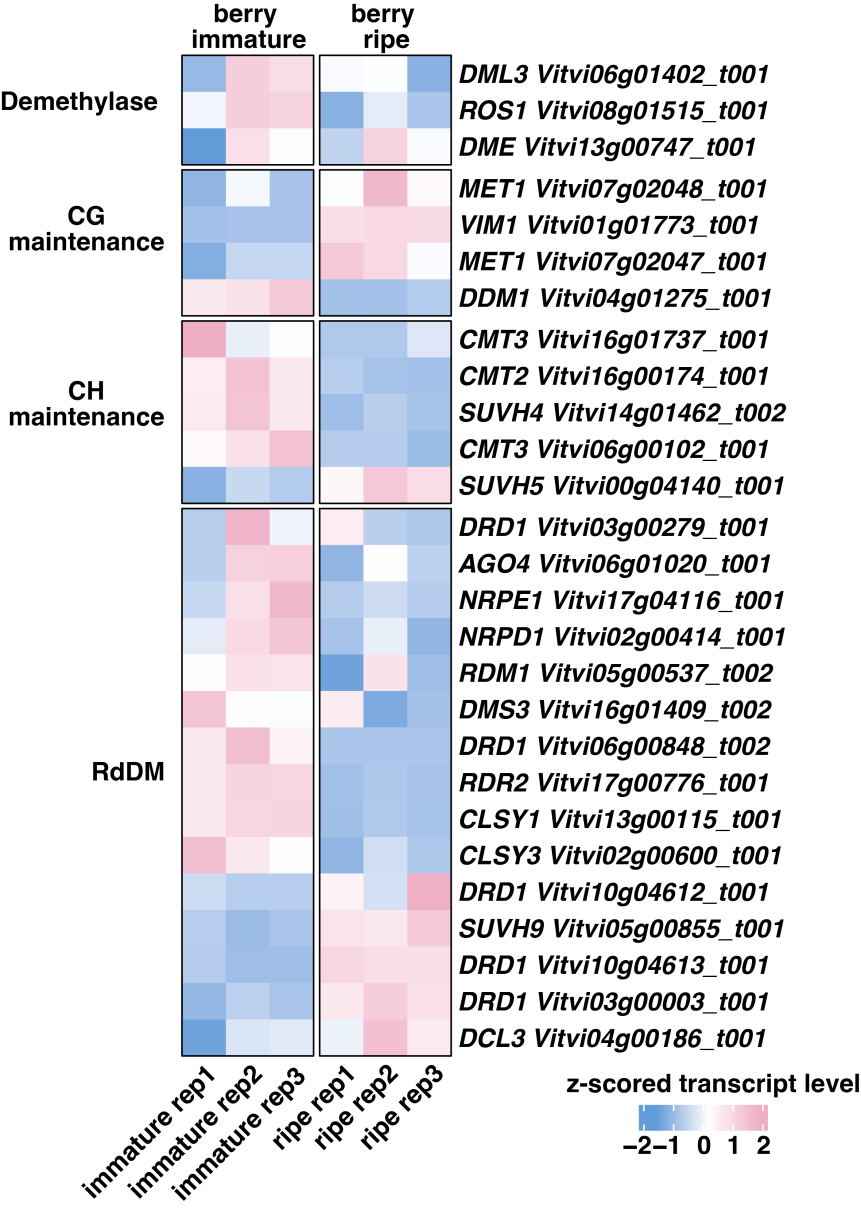


Figure S8. Relative transcript levels of genes involved in DNA methylation pathways in the whole berry of 'Wink' at immature and ripe stages.

The heatmap displays transcript level of genes associated with DNA demethylation, DNA methylation maintenance, and *de novo* DNA methylation processes.


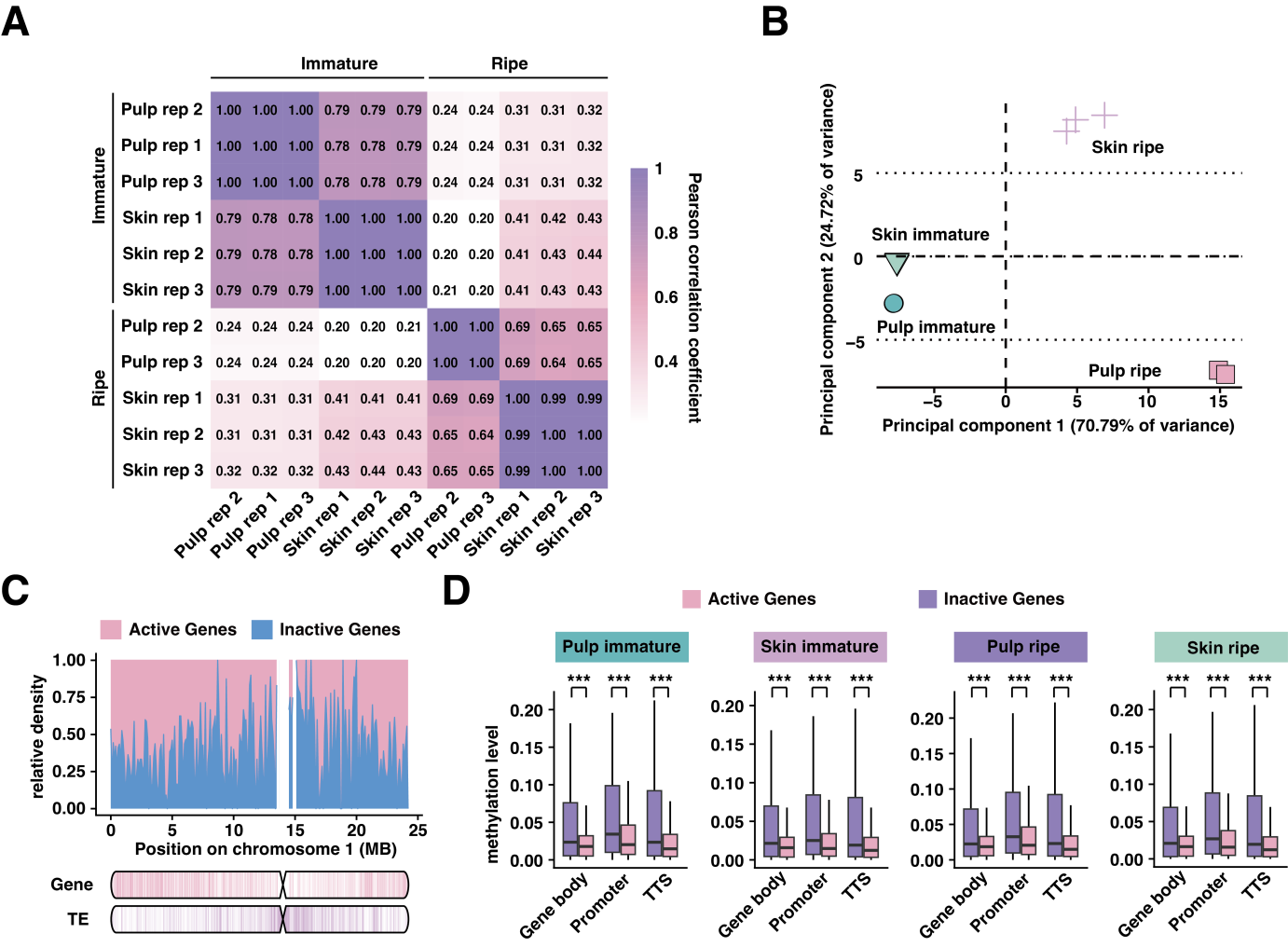


Figure S9. Association between DNA methylation and gene expression during grape fruit ripening.

(A) Pearson correlation coefficients matrix of transcriptome profiles across biological replicates of pulp and skin tissues at immature and ripe stages.

(B) Principal component analysis (PCA) of transcriptomes, illustrating tissue- and stage-specific clustering.

(C) Chromosomal distribution of active genes (FPKM ≥1) and inactive genes (FPKM <1) on chromosome 1. Active genes (pink) are enriched in gene-rich chromosome arms, whereas inactive genes (blue) localize to pericentromeric regions with high transposable element (TE) density.

(D) Methylation patterns of active and inactive genes in the gene body, promoter, and regions 2 kb downstream of the transcription termination site (TTS). Statistical comparisons were performed using a two-sided Wilcoxon rank-sum test. Asterisks denote significant differences (***p < 0.001).


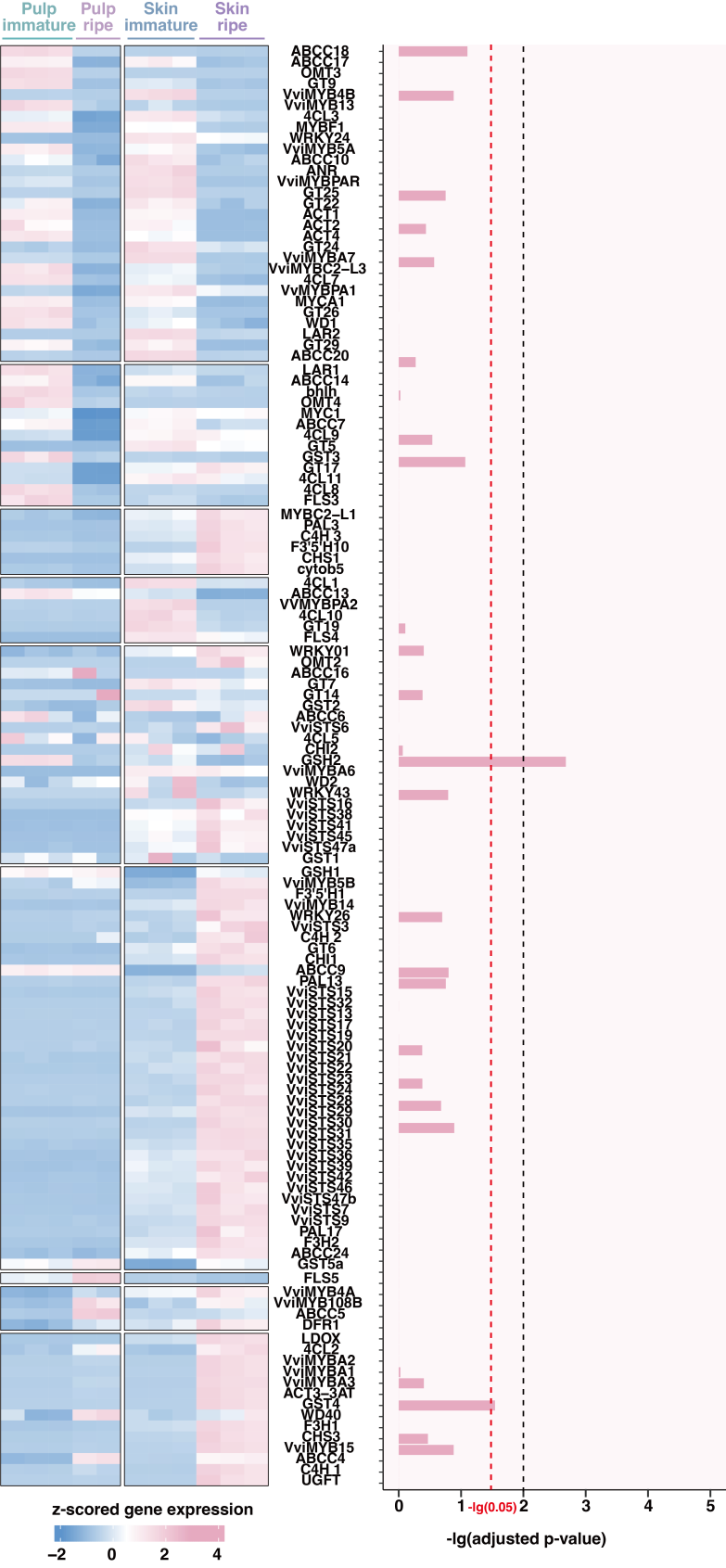


Figure S10. Association between DNA methylation and gene expression in the skin and pulp of 'Cabernet Sauvignon' at immature and ripe stages for genes involved in anthocyanin biosynthesis.

Promoter regions (2 kb upstream of transcription start sites) were divided into 20 bins of 100 bp each. The bin with the largest methylation difference was used to represent the DNA methylation level of each gene. Correlation analysis was then performed between methylation levels and gene expression to assess potential epigenetic regulation of anthocyanin biosynthesis during berry development.


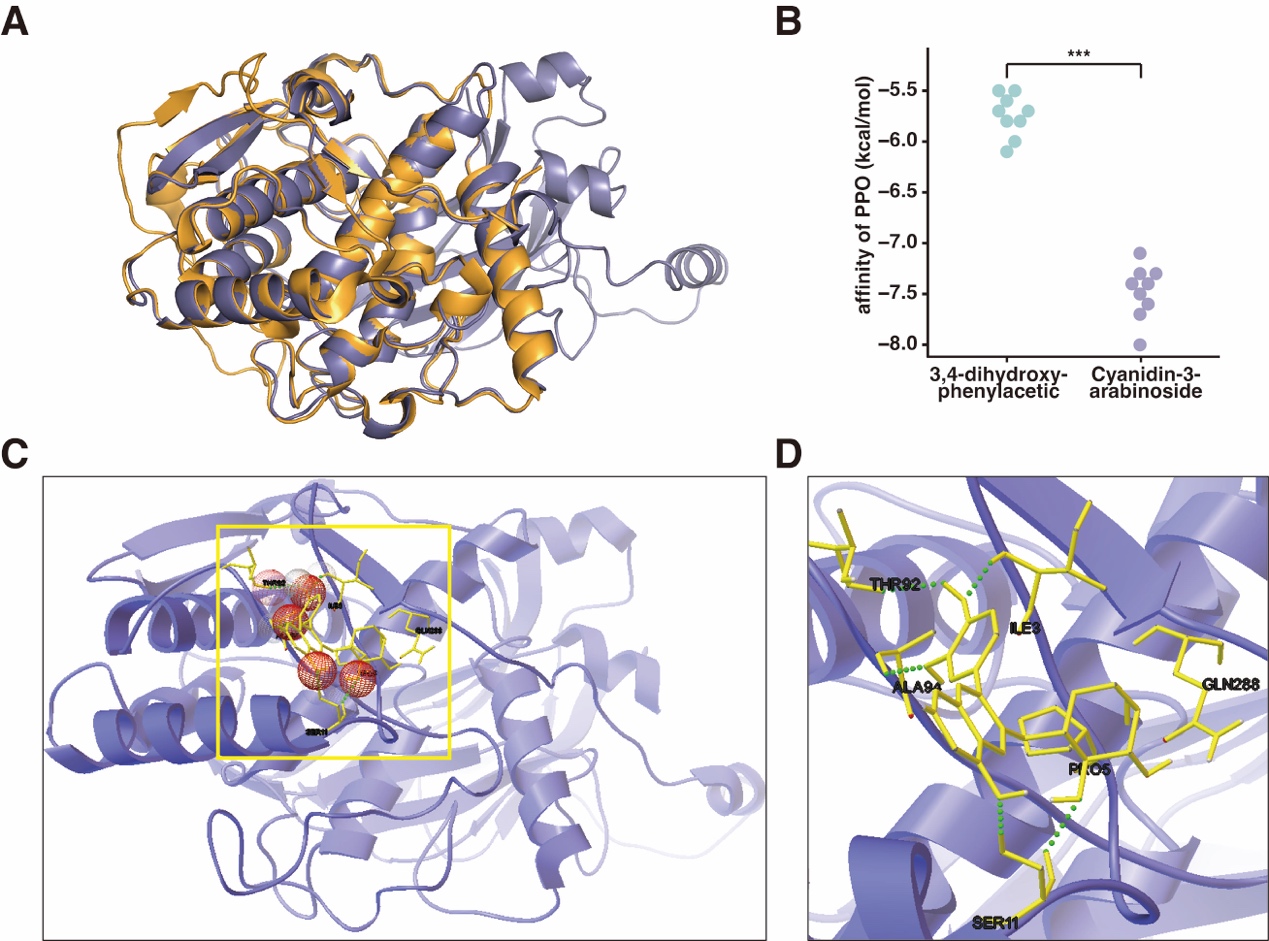


**Figure S11. Molecular docking analysis of the interaction between Vitvi10g04287-encoded polyphenol oxidase (PPO) and cyanidin-3-arabinoside.**

1. Structural homology between the AlphaFold3-predicted Vitvi10g04287-encoded PPO model (purple) and the crystal structure of Vitis vinifera (Grenache) PPO (PDB: 2P3X, orange), demonstrating high conformational similarity and model reliability. The two structures show high conformational similarity, with a root mean square deviation (RMSD) of **0.460 Å**.
2. Binding energy distributions for PPO (Vitvi10g04287) with cyanidin-3-arabinoside (mean = −7.5 kcal/mol, range = −8.0 to −7.1 kcal/mol) and the control 3,4-dihydroxyphenylacetic acid (3,4-DHPA; mean = −5.7 kcal/mol, range =−6.1 to −5.5 kcal/mol). A two-tailed t-test confirmed significant differences in binding energy (*** represents p < 0.001), highlighting stronger specific interactions with the anthocyanin.
3. Molecular interaction network between cyanidin-3-arabinoside (yellow) and PPO (purple), visualized via key residue engagements in the active site.
4. Detailed hydrogen bond formation (green dashed lines) between cyanidin-3-arabinoside and PPO at residues Ile3, Ser11, and Ala94.
