# Supplementary figures and images for: The grape berry methylome reveals tissue-specific features associated with metabolism in ripening
Source: Hortic Res. 2025 Sep 17;12(12):uhaf238. doi: 10.1093/hr/uhaf238 (PMC12682070; doi:10.1093/hr/uhaf238)

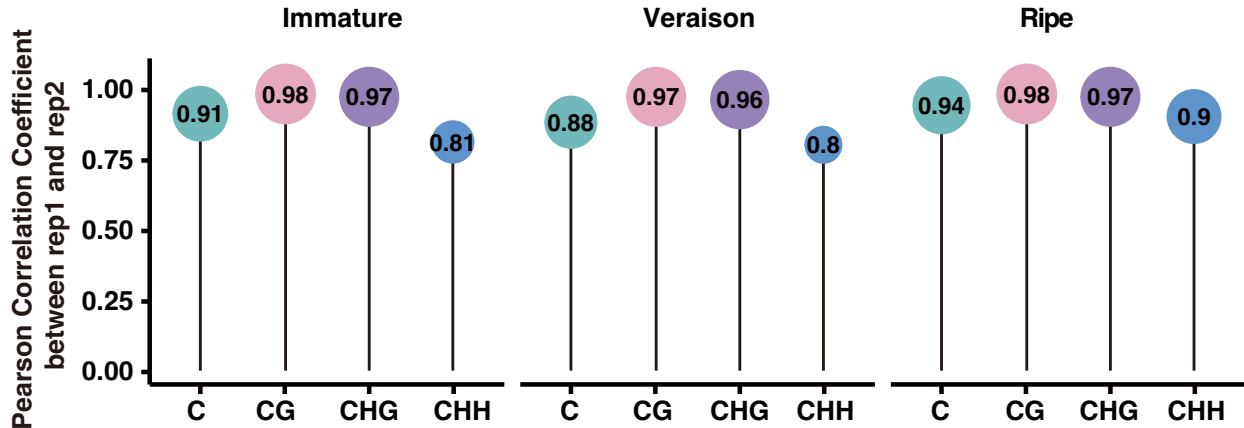

Supplement: Web_Material_uhaf238 [file web_material_uhaf238.zip › FigS1.pdf]

**A**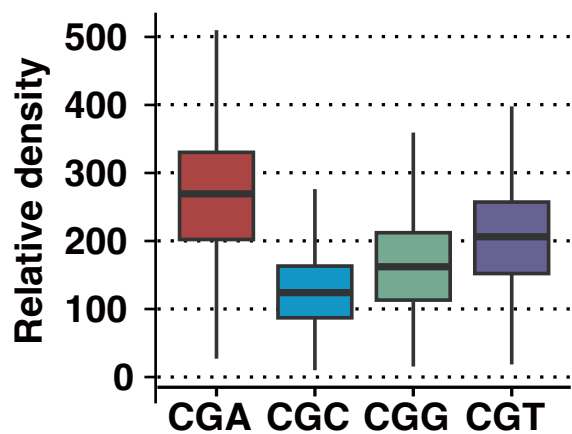**B**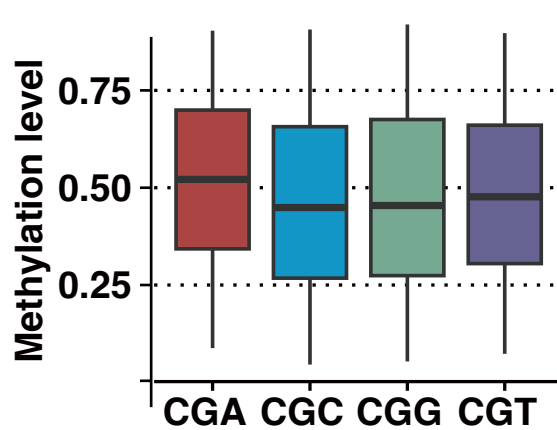**C**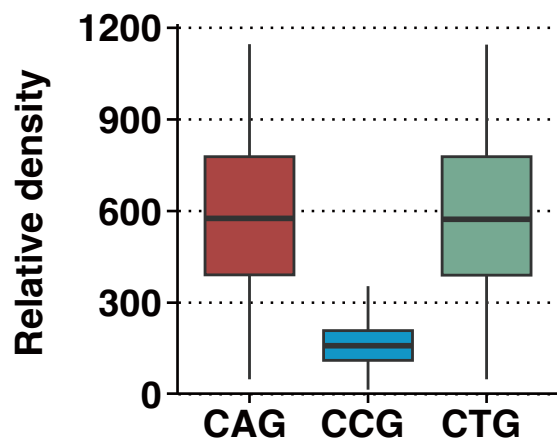**D**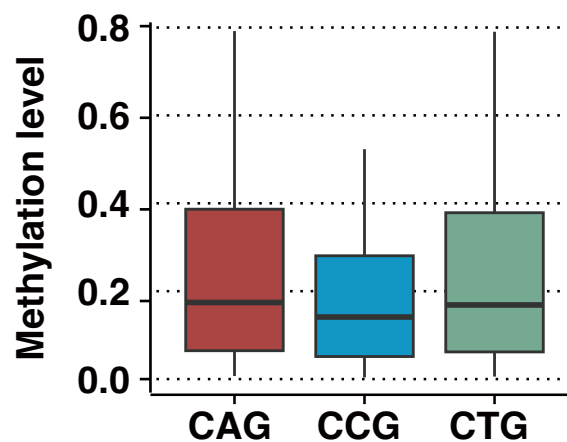**E**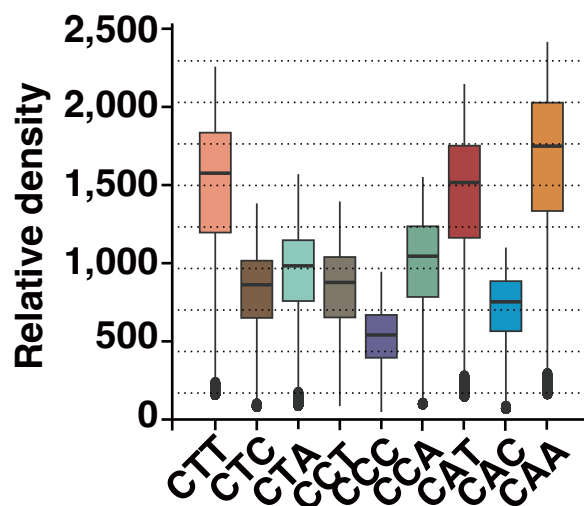**F**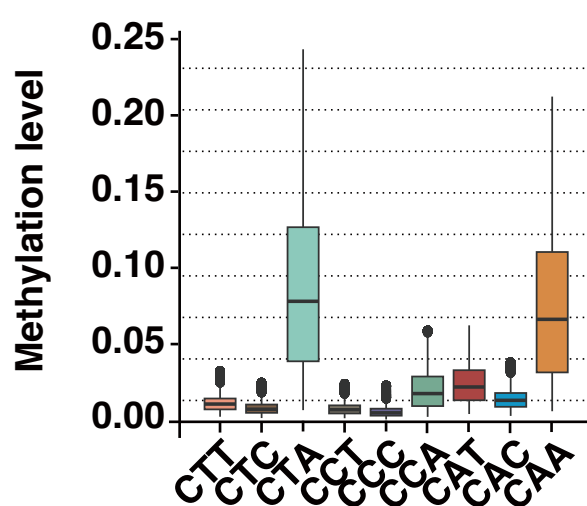

Supplement: Web_Material_uhaf238 [file web_material_uhaf238.zip › FigS2.pdf]

**A**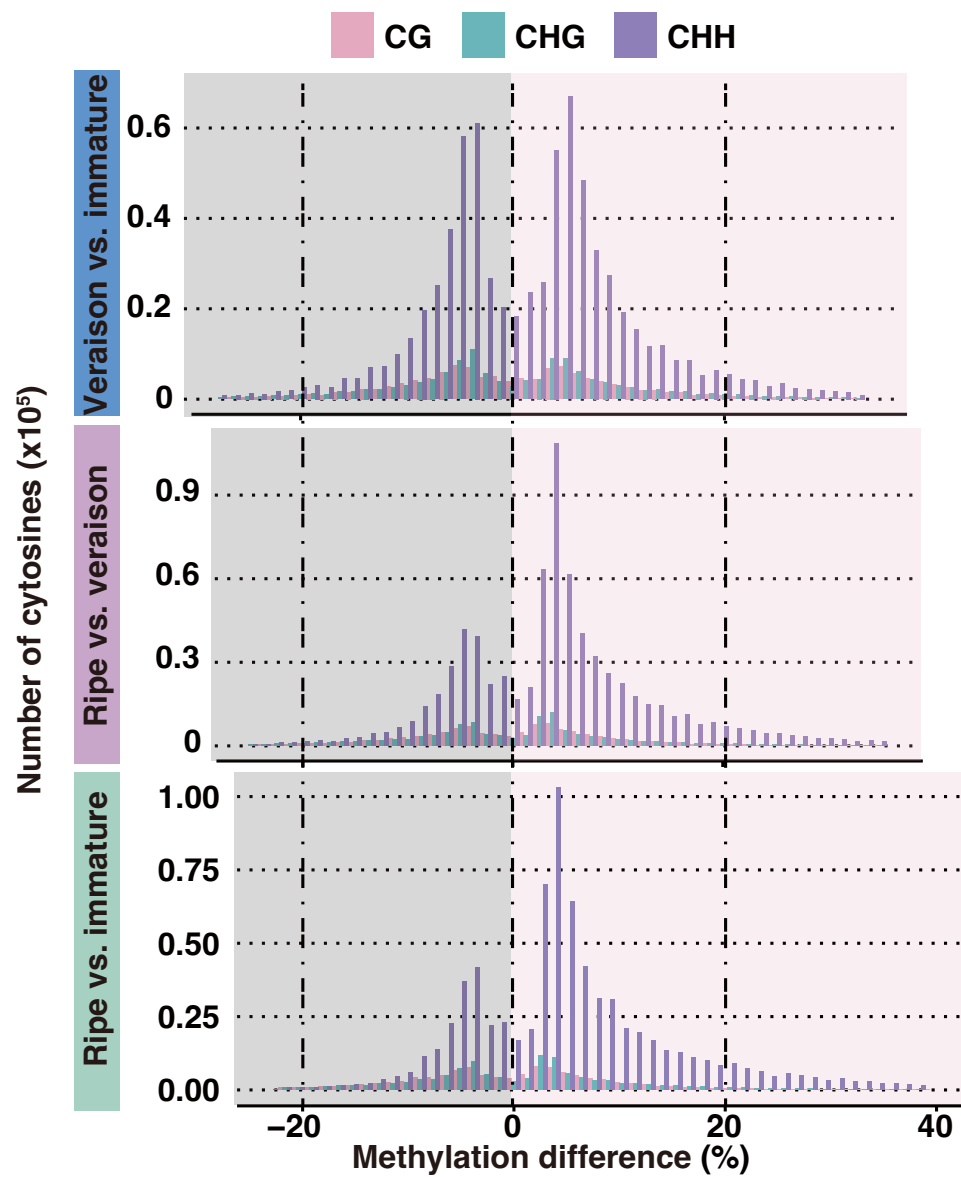**B**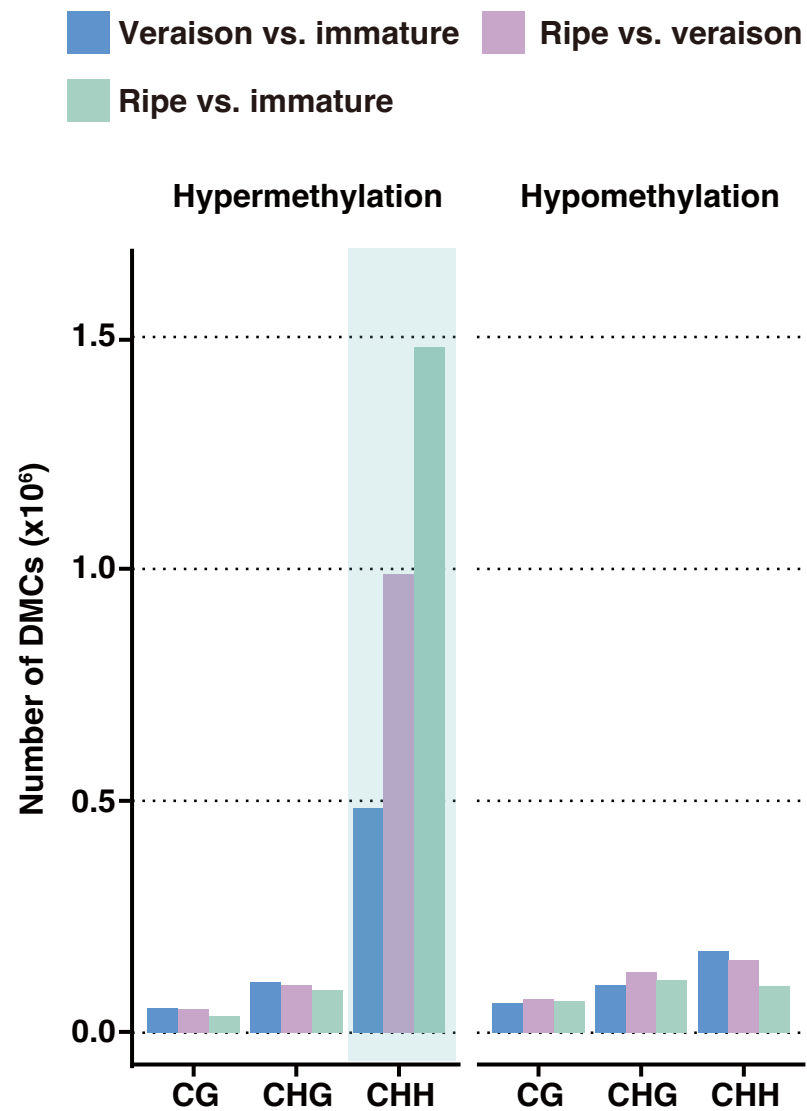

Supplement: Web_Material_uhaf238 [file web_material_uhaf238.zip › FigS3.pdf]

**Fruit Set**

**Veraison**

**F + 6W**  
**6 weeks after fruit set**

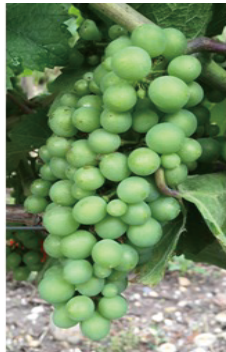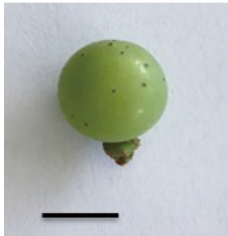

**V + 3W**  
**3 weeks after veraison**

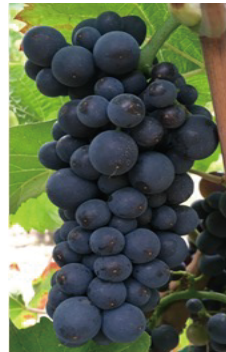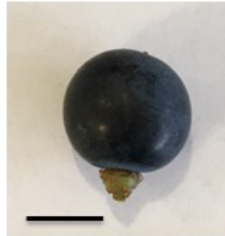

**'Cabernet  
Sauvignon'**

Supplement: Web_Material_uhaf238 [file web_material_uhaf238.zip › FigS4.pdf]

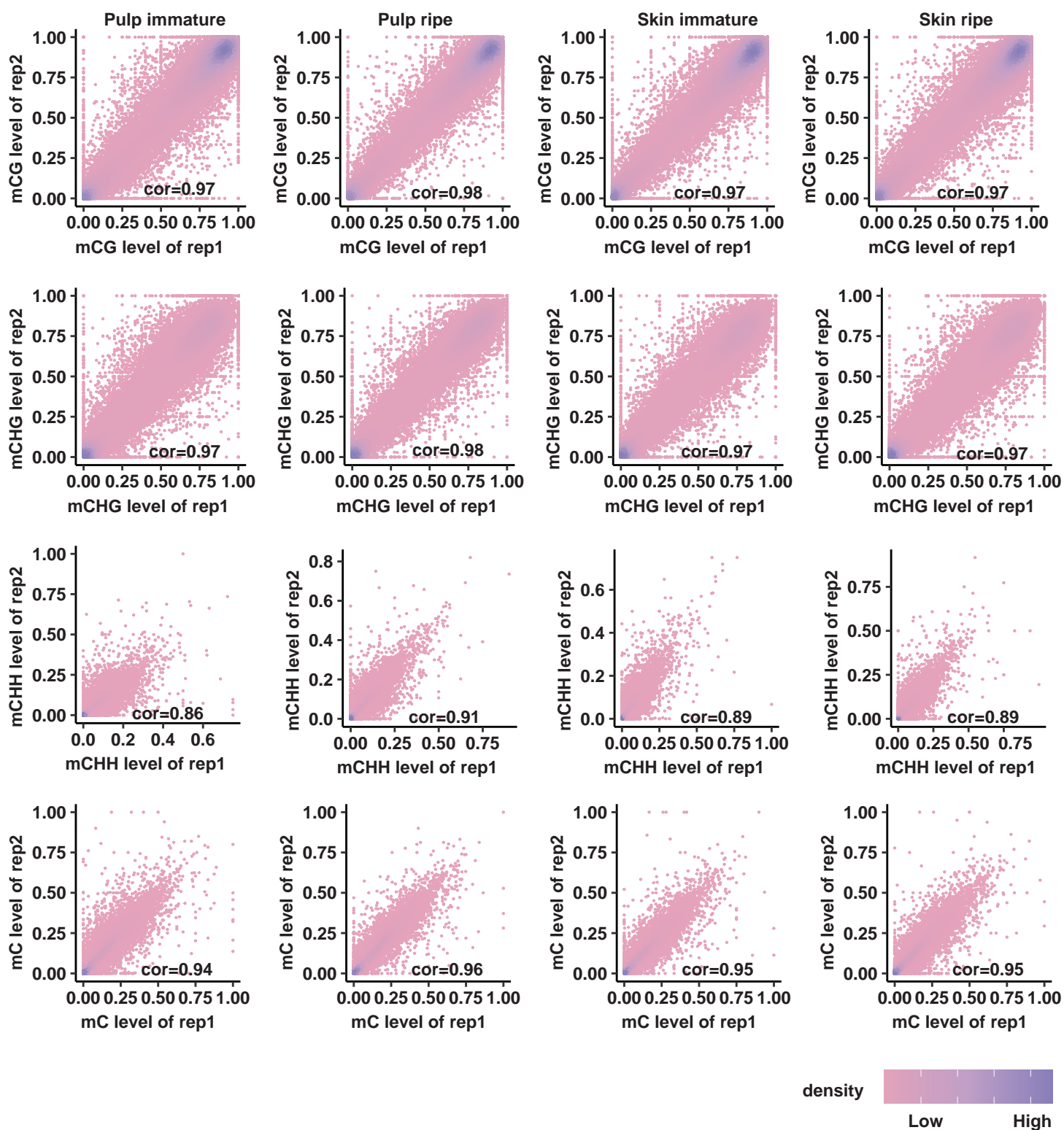

Supplement: Web_Material_uhaf238 [file web_material_uhaf238.zip › FigS5.pdf]

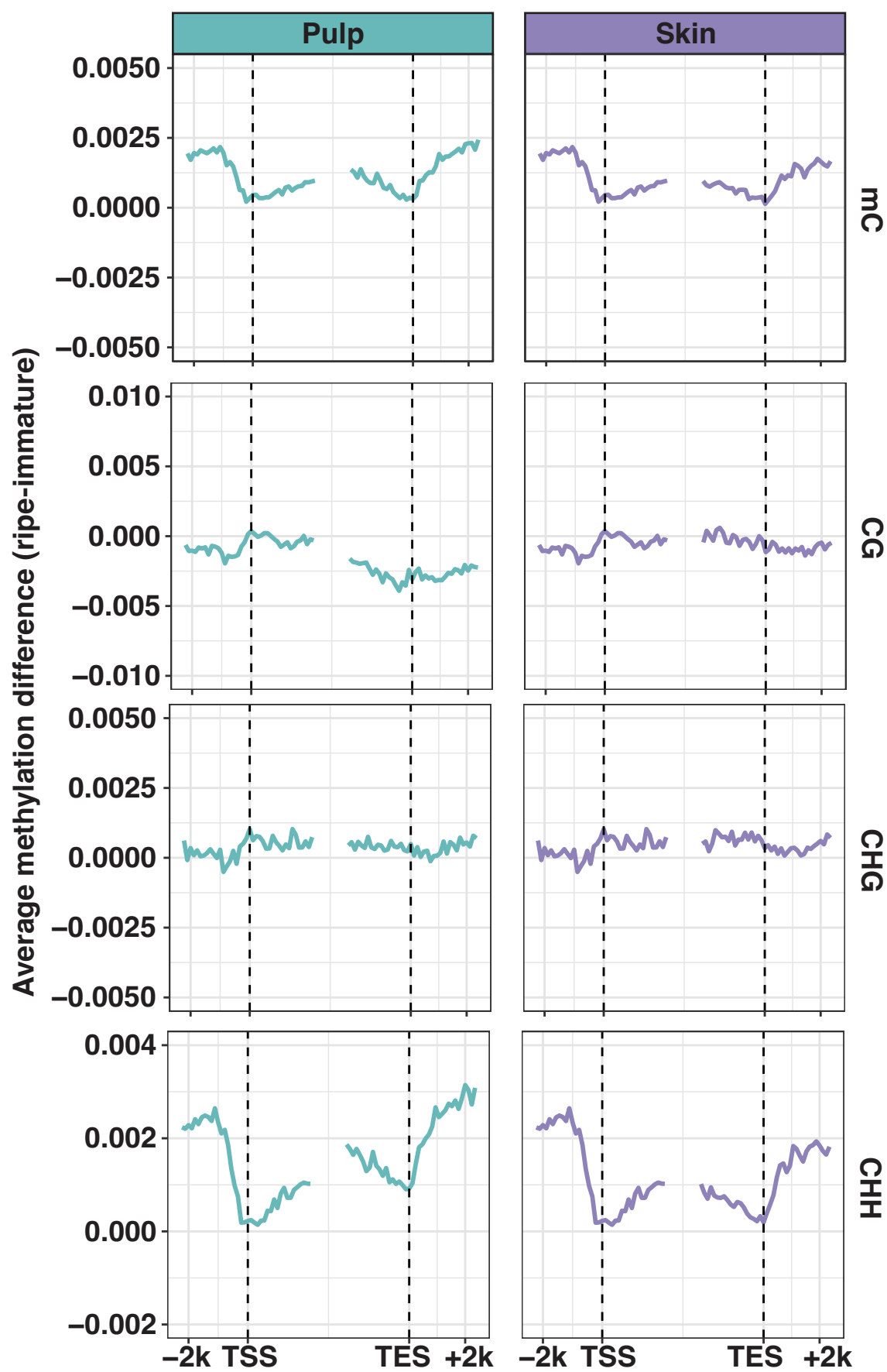

Supplement: Web_Material_uhaf238 [file web_material_uhaf238.zip › FigS6.pdf]

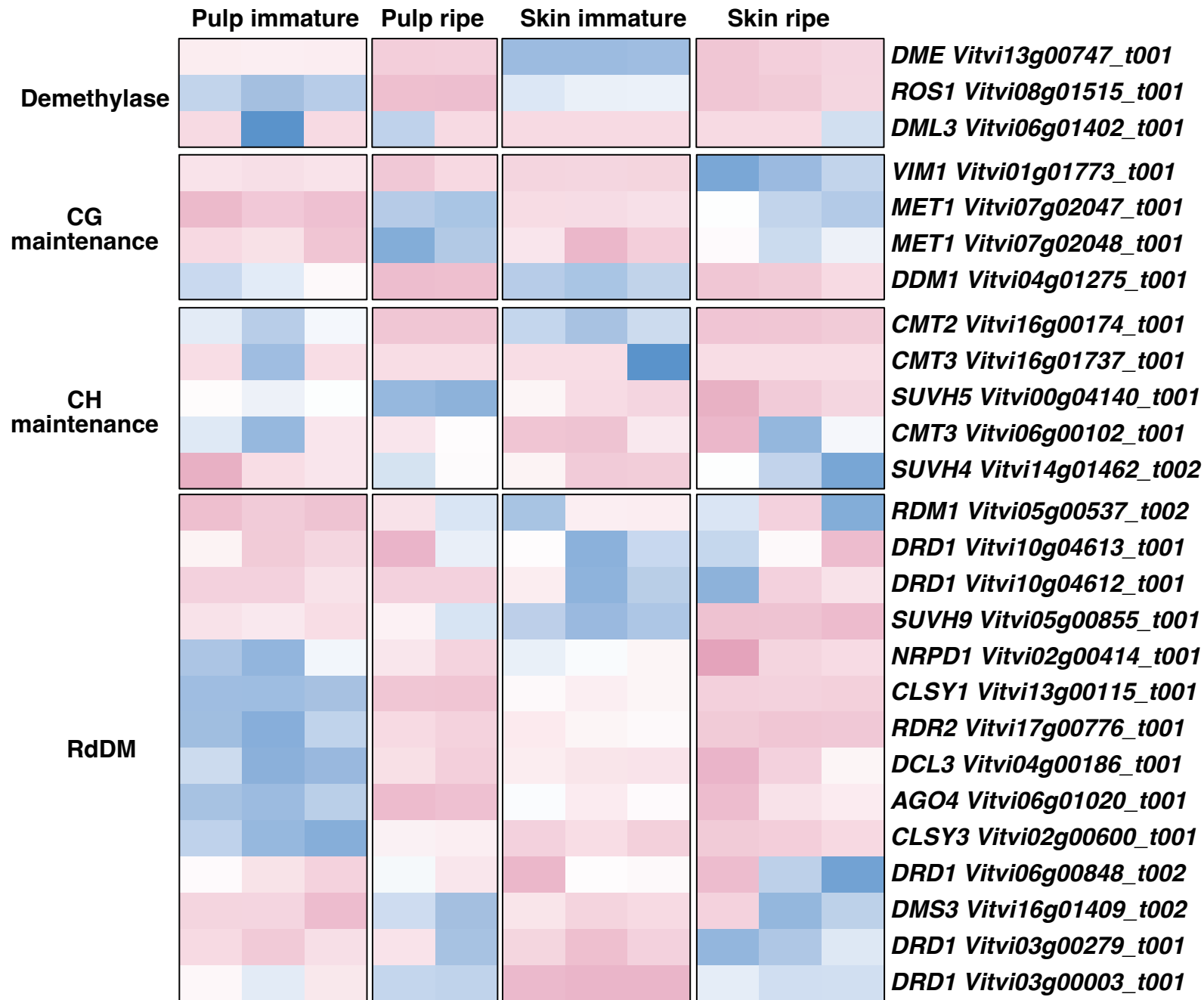

z-scored transcript level

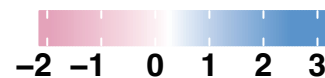

Supplement: Web_Material_uhaf238 [file web_material_uhaf238.zip › FigS7.pdf]

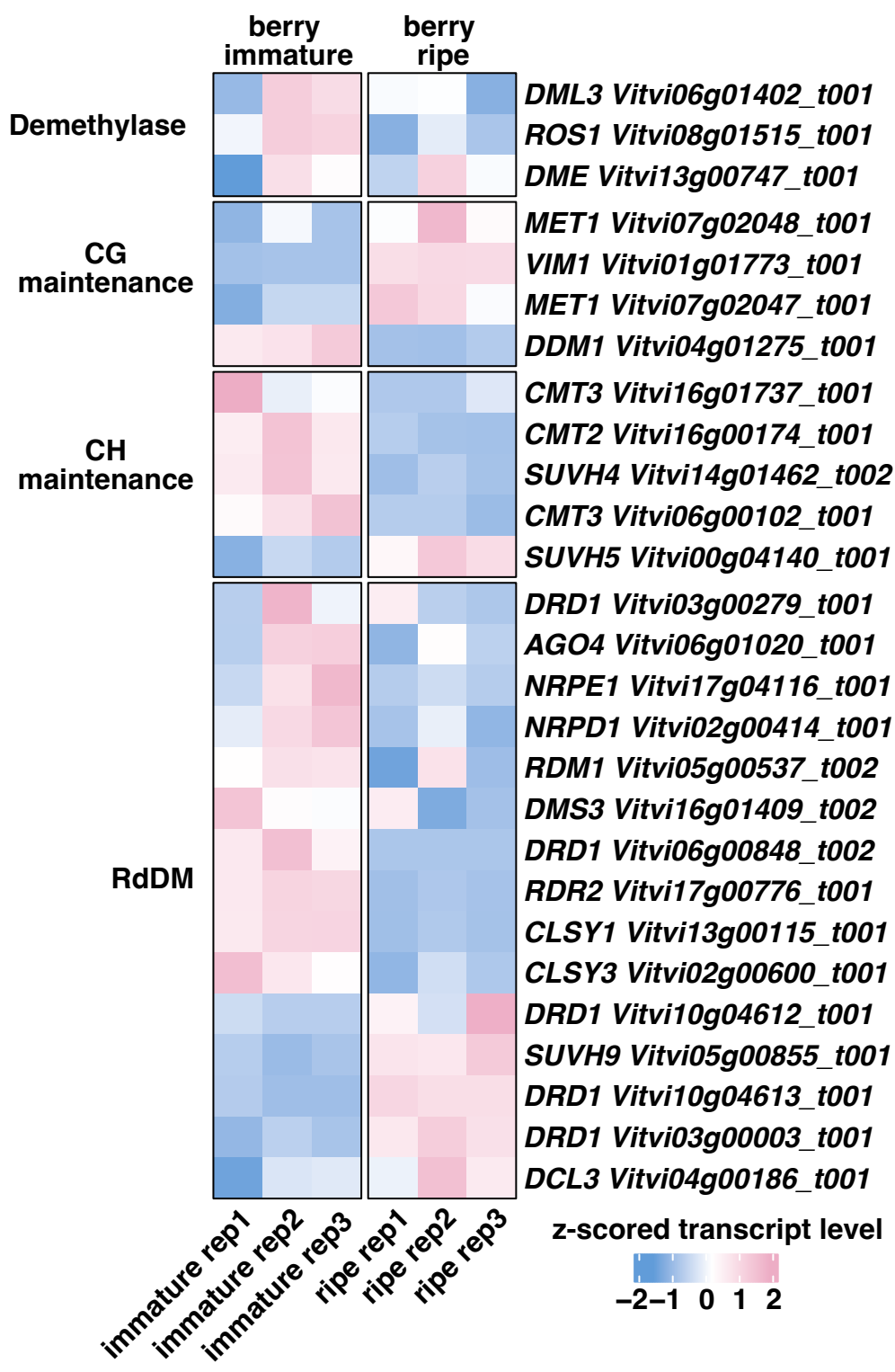

Supplement: Web_Material_uhaf238 [file web_material_uhaf238.zip › FigS8.pdf]

A

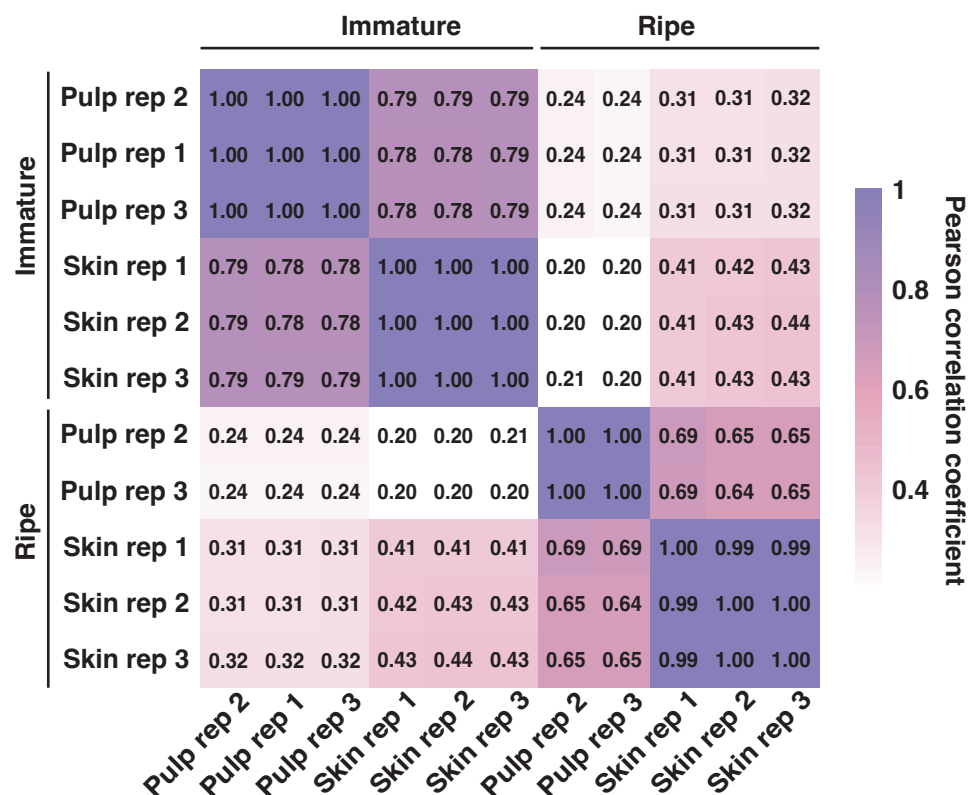

B

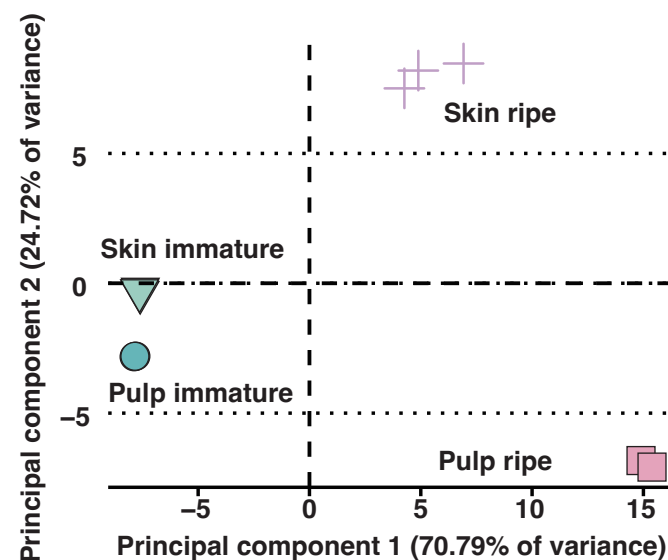

C

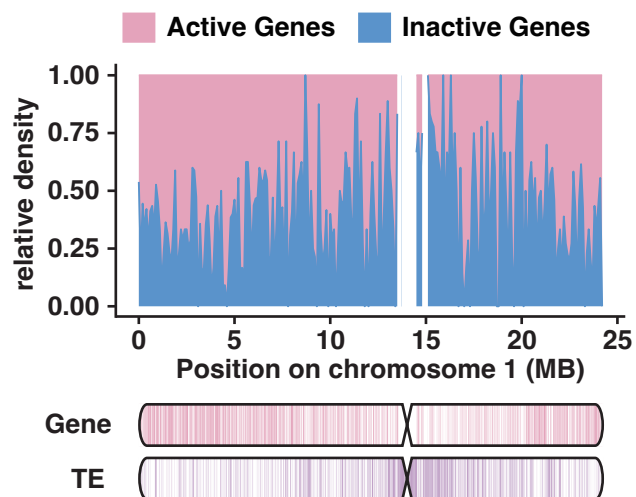

D

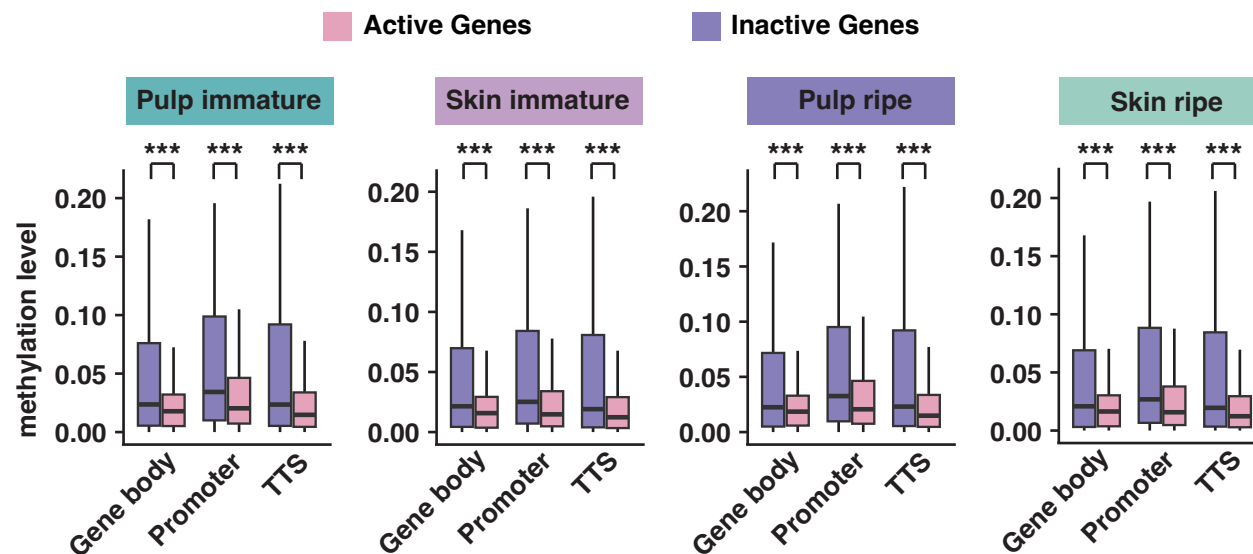

Supplement: Web_Material_uhaf238 [file web_material_uhaf238.zip › FigS9.pdf]

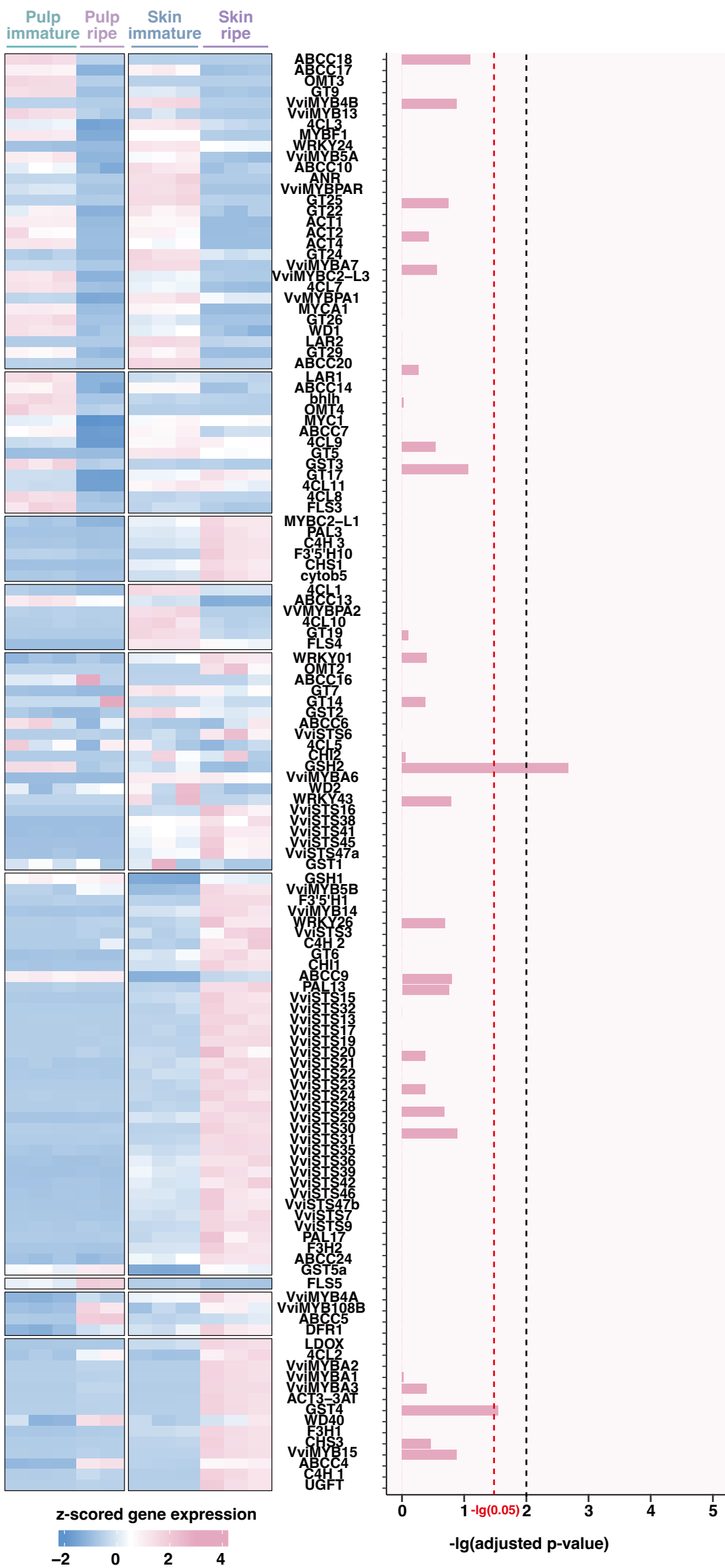

Supplement: Web_Material_uhaf238 [file web_material_uhaf238.zip › FigS10.pdf]

**A**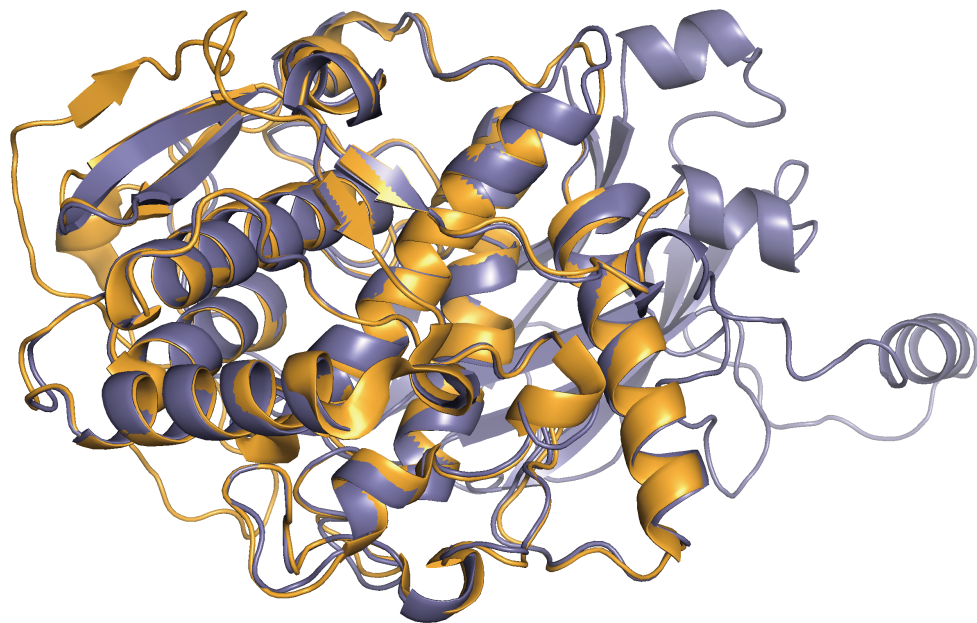**B**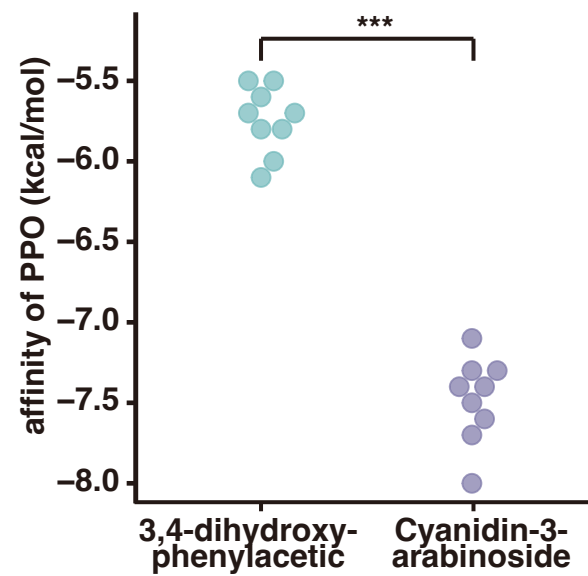**C**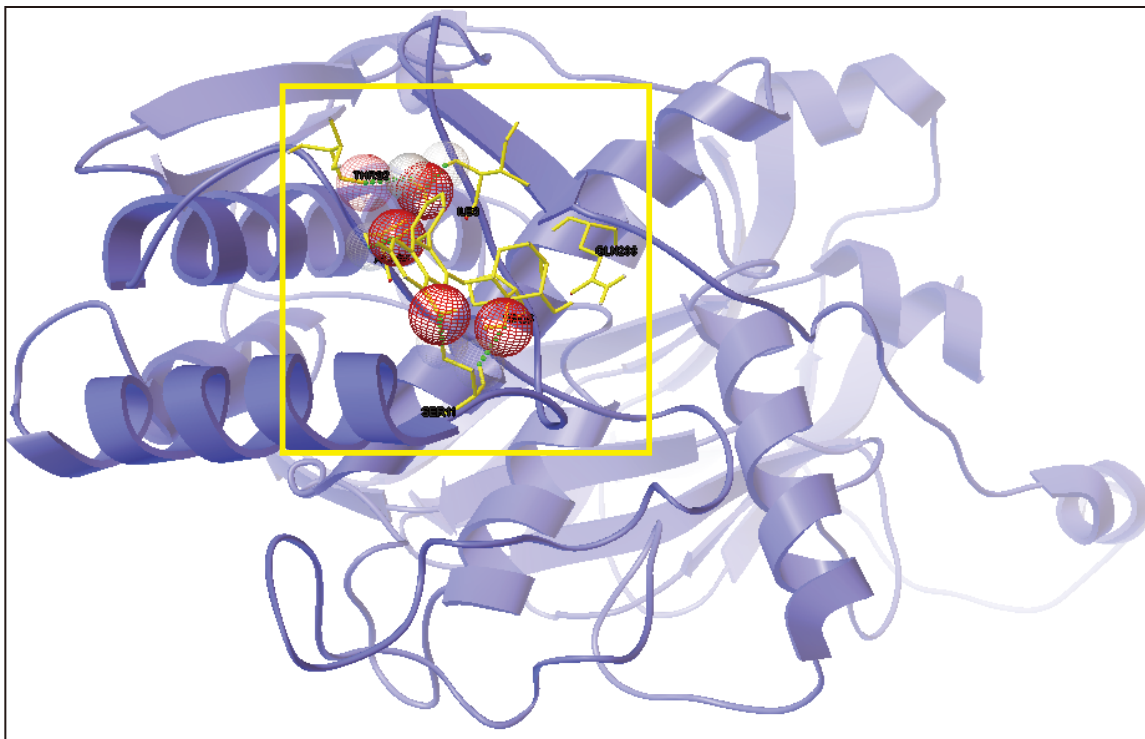**D**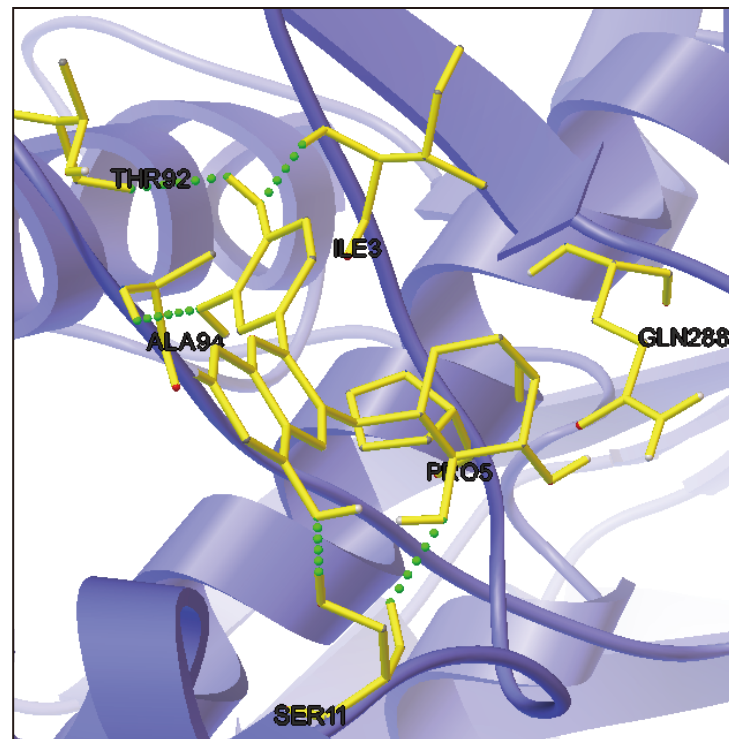

Supplement: Web_Material_uhaf238 [file web_material_uhaf238.zip › FigS11.pdf]
